# Association between coach-athlete relationship and athlete engagement in Chinese team sports: The mediating effect of thriving

**DOI:** 10.1371/journal.pone.0289979

**Published:** 2023-08-17

**Authors:** Song Gu, Wenxuan Peng, Feiyue Du, Xuemo Fang, Zhixun Guan, Xiaolong He, Xulu Jiang

**Affiliations:** 1 College of Physical Education and Health Sciences, Zhejiang Normal University, Jinhua, China; 2 College of Teacher Education, Zhejiang Normal University, Jinhua, China; Ahvaz Jundishapur University: Ahvaz Jondishapour University of Medical Sciences, ISLAMIC REPUBLIC OF IRAN

## Abstract

**Background:**

Coach-athlete relationship (CAR), thriving and athlete engagement are important psychological variables that affect sports performance. On the basis of self-determination theory, this study constructs a mediation model to examine the influence of CAR on athlete engagement and the mediating effect of thriving between them.

**Methods:**

This cross-sectional study involves a questionnaire survey with 287 Chinese active athletes (M = 19.63, SD = 2.53) aged 14–26 years (64.5% male, 35.5% female) from eight sports. CAR, thriving and athlete engagement were assessed using the CAR Questionnaire, the Thriving Scale, and the Athlete Engagement Questionnaire, respectively.

**Results:**

CAR and its dimensions can significantly and positively predict athlete engagement, complementarity, commitment, and closeness, accounting for 35.1%, 34.6%, and 30.4% of the cumulative variance in dominance analysis, respectively. The direct and indirect paths show that CAR affects athlete engagement through the mediating effect of thriving. The mediating effect model has a good fit and indirect effects account for 56.9% of the total effects.

**Conclusion:**

The effect of CAR on athlete engagement reflects a practical application of interpersonal dynamics in competitive sports to a certain extent. The following suggestions can be used to improve athlete engagement. First, setting common goals, emphasizing mutual cooperation, and building trust and support, promote coaches and athletes to have a higher sense of commitment and complementarity to each other, thereby helping improve athlete engagement. Second, meeting the vitality and progress needs of athletes effectively mobilizes CAR resources to promote athlete engagement, which can be manipulated by cultivating closeness, commitment, and complementarity. Third, to ensure the athletes’ sports state and mental health, the sports team should focus on the cultivation of athletes’ capacities to thrive and internally form a dynamic and positive sports atmosphere in their team. In the future, we can track and compare the influence of the improvement of CAR on thriving and athlete engagement can be tracked and compared from the dual perspectives of coaches and athletes.

## Introduction

The rise of positive psychology have led researchers to gradually shift their focus from solving the negative individual problems to promoting positive psychology. An example of a group that faces high psychological loss are athletes. Maintaining a thriving state is beneficial to their performance and career development [1]. Engagement is a lasting and positive cognitive and emotional experience for athletes [2], which plays a positive role in improving athletes’ performance and ensuring their mental health [3,4]. How to maintain or improve the level of athlete engagement has become a problem that coaches and athletes need to pay attention to. At present, the antecedent variables of athlete engagement is mainly from the perspective of intrinsic motivation, with insufficient emphasis on the interpersonal environment as a contributing factor. As the direct leader of the sports team, coaches’ behavior is the main external factor that motivates athletes to actively participate in training and competition and improve their competitive ability. In addition, coaches are an important source of social, emotional, and informational support for athletes, and their effects on athlete engagement cannot be ignored. The coach-athlete relationship (CAR) is a two-way interactive process. The quality of interaction between coaches and athletes adjusts their perception of relationship costs and benefits. Athlete engagement includes cognitive and emotional components. From these components, athlete engagement is bound to be affected by the quality of CAR. Therefore, this study aims to examine the influence of CAR, on athlete engagement, providing insights and support for improving athlete engagement from coaches and team development strategies. In addition, given the different ways in which athletes and coaches interact in individual and team sports, this study focuses on team sports to yield representative results.

### Athlete engagement and CAR

In line with the principles of positive psychology, organizational psychologists have suggested that engagement is the conceptual opposite of burnout, characterized by a permanent filled with positive emotions and motivations [5]. Lonsdale (2006) introduced this concept into the realm of sports, highlighting that athlete engagement reflects a lasting and positive cognitive and emotional experience in sports [2–4]. The author also determined that athlete engagement is the best link among individual characteristics, sports factors, and sports performance [3]. Given these concepts, Lonsdale (2006) proposed the following as the main characteristics of athlete engagement: a) confidence, which is the “belief in one’s ability to attain a high level of performance and achieve desired goals;” b) dedication, which is “a desire to invest effort and time toward achieving goals one views as important;” c) vigor, which is as “a sense of physical and mental liveliness;” and d) enthusiasm, which is characterized by “feelings of excitement and high levels of enjoyment.”

Different from sensory indicators, such as sports satisfaction, athlete engagement intuitively reflects the positive experience of individual cognition [3]. Furthermore, athlete engagement is also the best way to prevent athletes from experiencing burnout symptoms [5]. Given the important influence of athlete engagement on performance, understanding the formation mechanism of athlete engagement is highly valuable in improving athletes’ competitive performance.

Existing research on athlete engagement primarily adopts perspectives from work engagement, with verified antecedent variables such as motivation [6], gratitude [7], coping style [8], social support [9,10], understanding care [11], and coaches’ leadership behavior [12]. Among the individual factors, motivation has the most significant influence [13–15], wheres social support holds the most important role among environmental factors [9]. Therefore, combining environment and motivation offers an ideal framework for investigating the formation mechanisms of athlete engagement.

While exploring the relationship between cohesion and athlete engagement, Song (2022) emphasized that coaches, as leaders, are responsible for implementing strategies for improving the team environment [16]. As the most influential figure in a team, coaches’ influence on athletes is directly related to their performance and career development [17–21]. This influence mainly includes leadership behavior [12,22,23], leadership style [24–27], coaching ability [28], and CAR [17,20,29–31]. Particularly, the CAR, as an important interpersonal relationship throughout athletes’ career, is an important source of social support for athletes [9,32–35]. It reflects the emotional, mental, and behavioral interactions between coaches and athletes [31]. Numerous examples of successful CAR exists such as Olympic champion 110-meter hurdler Liu Xiang and his coach Sun Haiping; swimmer Michael Phelps and his coach Bowman, freestyle skier Eileen Gu and her coach Brad Prosserd, and other CARs that synergize to create excellent sports careers.

At present, two theories can explain CAR. The first theory follows the motivational model to explore the influence of coaches’ autonomous support behaviors on athletes’ self-determined motivation. Mageau and Vallerand (2003) suggested that when coaches exhibit autonomous support behavior, they can consider issues from the athletes perspectives and respect their personal feelings. This approach offers relevant information and opportunities for athletes to practice their independent choices and diminishes the need for coercion, thereby promoting the satisfaction of athletes’ autonomy needs, performance requirements, and relationship necessities. Ultimately, this approach stimulates athletes’ self-determined motivation [17].

The second theory is the interpretation framework based on the concepts of belonging needs and intimacy, such as La Voi’s conceptual model, Poczwardowski’s conceptual model, and CAR’s 3+1Cs model [32]. Particularly, the CAR’s 3+1Cs model proposed by Jowett (2007) is the earliest and most widely adopted [33]. Originating from the emotion, cognition and behavior within the CAR structure, the author proposed the following corresponding characteristics: closeness, which signifies the emotional bond that athletes and coaches attribute to their relationship (e.g., trust, likability, and respect); commitment, which implies the athletes and coaches’ intention to maintain their athletic relationship and optimize its benefits; and complementarity, which refers to how athletes and coaches interact in terms of affiliation (e.g., a friendly, responsive attitude from an athlete may elicit a similar response from a coach), as well as reciprocal dominance and submission (e.g., a coach instructs and an athlete executes).

In Jowett’s research, all three characteristics can be evaluated through the direct perspective, the meta perspective, and the co-orientation of coaches and athletes [24]. Given that dyadic co-orientation is difficult to operate using mathematical statistics, most existing studies tend to define CAR from the direct or meta perspective of coaches and athletes [11]. Generally, the 3+1Cs model highlights the reciprocal interaction between athletes and coaches, revealing the two-way communication process between them from a dynamic perspective. This model encompasses the three levels of cognition, emotion, and behavior, and thoroughly exposes the interaction mode between the relationship’s subjects.

At present, the latter variables of CAR include aspects such as sports performance satisfaction [34,35], motivational atmosphere [36], achievement goals [37], self-determined motivation [18], passion [38], subjective wellbeing [11], cohesion [39], resilience [40], collective efficacy [35], interpersonal conflict [41], and burnout [42]. However, the influence of CAR on athlete engagement has not been extensively explored. Recent studies have discovered that when students perceive a positive relationship with their teachers, they exhibit higher learning engagement [43]. Similarly, in organizational behavior, when members perceive a good relationship quality with their leader, employees feel energetic and engaged in their work [44]. Moreover, CAR has has a negative correlation with sports burnout; hence, as the opposite of burnout [45,46], athlete engagement may be positively affected by CAR. Thus, the following hypothesis is proposed:

Hypothesis 1 (H1). CAR and its dimensions can positively predict athlete engagement significantly.

### Athlete thriving as a mediator

As Bollen (1989) indicated, “when the mediator is included, the understanding of the process will be more complete” [47]. Thus, SDT is considered a potential foundation for studying the requirements of athlete engagement. According to SDT, the social environment comprises cognitive, emotional, and behavioral factors that influence individuals through autonomous motivation (e.g., identified regulation, integrated regulation, and internal motivation) [48].

In this study, CAR and athlete engagement correspond to the “social environment” and “cognitive, emotional, and behavioral results,” respectively, whereas thriving corresponds to “autonomous motivation”. Thriving is strongly connected to autonomous motivation and represents a dynamic, positive psychological state or feeling that comprehensively reflects individuals’ growth, development, and success [49]. Individuals who are thriving experience growth and momentum, characterized by a sense of feeling energized and alive (vitality) and a belief that they are continually improving and becoming better at what they do (learning) [50]. In the field of organizational behavior, individuals with high thriving exhibit higher job satisfaction [51], organizational loyalty [52], physical and mental health [53], and reduced job burnout [54]. As a result, they are likely to contribute to improved individual job performance. Therefore, developing athletes’ thriving effectively improve their performance and health.

Thriving is frequently applied in the field of work. Carver (1998) described thriving as the psychological experience of growth in a positive capacity that energizes and enlivens (i.e., constructive or forward direction) [55]. Spreitzer (2012) first proposed that thriving includes vitality [56], which is the state of an individual filled with energy and enthusiasm for work, and learning, which is the feeling of improving individual ability and self-efficacy the mastery of knowledge and skills [57,58]. The two dimensions are interdependent and influence the affective (vitality) and cognitive (learning) aspects of the psychological experience of personal growth [50]. Thriving is an individual’s state in a particular situation and is not a permanent disposition [59]. Therefore, thriving varies with changes in situation and time, but will not suddenly appear in its completely opposite state or form [60].

In a relatively short and continuous time frame, thriving is considered as a measurable and relatively stable state. In the field of sports, Gucciardi (2017) first introduced the concept of thriving and investigated the relationship between coaches’ controlling behavior and athletes’ thriving, discovering that coaches’ intimidation and threatening behaviors inhibit athletes from thriving [61]. Brown (2018) then examined the thriving of elite athletes and suggested that thriving helps improve athletes’ internal motivation and self-efficacy, thereby maintaining high levels of sports performance and promoting positive and healthy development. Generally, thriving reflects the track of individuals’ positive development, which is benificial for athletes who frequently face pressure and challenges [62].

Meeting basic psychological needs is considered crucial for individuals’ thriving development [1]. According to SDT, when the three basic needs (autonomy needs, competence needs and relatedness needs) are met, people obtain the best motivation for personal development [63,64].

The external environment plays a crucial role in meeting basic psychological needs. For athletes, the relationship between them and their coaches is at the core of external environmental factors [65]. In the 3Cs model, closeness and commitment are positively related to the competence and autonomy needs of athletes [66,67]. Brown (2018) used the elite athlete thriving model and argued that the antecedents of athletes’ thriving include high-quality relationships, described as good and effective interpersonal relationships formed among team members [62]. In summary, SDT and the elite athlete thriving model, which point to CAR explain the propensity for athletes to thrive in sports.

Only a few studies have focused on the relationship between thriving and athlete engagement. Thriving is associated with a series of positive happiness, learning and performance outcomes. For example, it significantly predicts the work engagement of organization members and is highly correlated with the subjective wellbeing of young athletes [1,68]. In other studies, subjective wellbeing predicts athlete engagement [69]. Podlog (2015) even demonstrated that athletes’ internal motivation has a significantly positive correlation with athlete engagement [13], with thriving being a psychological variable similar to self-efficacy and personal motivation [1]. In organizational behavior, the mediating role of the thriving has also received considerable scholarly attention [70,71]. In sum, CAR may affect athlete engagement through thriving. The interpretation framework of this effect is as follows, Good CAR provides environmental conditions to meet the basic psychological needs of athletes, and athletes then gain unity within the group and self-identity. Consequently, athletes’ autonomous motivations are stimulated, and are reflected in high vitality and initiative during training and competition. These positive emotional and cognitive experiences further promote athlete engagement in training and competition. Thus, the following hypothesis is proposed:

Hypothesis 2 (H2). CAR, as mediated by thriving, significantly and positively predicts athlete engagement.

## Materials and methods

This study was conducted according to the guidelines of the Declaration of Helsinki and approved by the Ethics Committee of Zhejiang Normal University (ZSRTZSRT2023001, 23 January 2023).

### Recruitment and participants

Mueller (1997) suggested that the ratio of sample size to the number of observed variables should be at least 10:1 to 15:1 for pure SEM analysis [72]; Rigdon (2005) argued that when the analysis sample of an SEM model is less than 200, the model fitting becomes unstable [72]. In view of the sample size of this study, the survey sample size is determined to be over 300.

From February 7 to 20, 2023, the researchers were divided into two groups and visited the locations of some sports training teams in Heilongjiang, Liaoning, and Zhejiang Provinces. A total of 358 provincial and national team players participated in the questionnaire survey. They were all active athletes in team sports (e.g., basketball, volleyball, and football), aged 14–26, and had more than three years of training experience. All questionnaire surveys were conducted after sports training sessions. The researchers initially obtained consent from the coaches and athletes for the research, explained the purpose and confidentiality of the research to them, and then had the athletes’ representatives from each sports team sign the informed consent form to ensure that the respondents understood the possible risks. To eliminate interference from coaches, the questionnaire was distributed by researchers under the organization of the sports team captain. The participants then completed the questionnaire, which took approximately 12 min, and returned it directly.

### Instruments

The questionnaires involved in this study were in Chinese language, there were three sections to the questionnaire. First, we made it clear that this survey was voluntary and anonymous. The questionnaire’s responses were only made available to the researchers; they could not be used for profit or for any other purpose. The second step is to collect basic information about the athletes. In the third part of this study, we considered the scale of the questionnaire. In this study, all questionnaire responses were scored on a five-point Likert scale, from strongly disagree (1) to strongly agree (5). The higher the score, the more recognized and accepted the item was. In detail, here are the details:

#### Coach-Athlete Relationship Questionnaire (CARQ)

The CARQ, compiled by Jowett and Ntoumanis [73] and translated and revised by Zhong Risheng and Wang Di [74], has been tested for its applicability among Chinese athletes and has good reliability and validity. The questionnaire has a total of 11 items, including three dimensions of closeness, commitment and complementarity. CAR scale has a good fit (χ^2^/df = 2.89, RMSEA = 0.08, SRMR = 0.05,CFI = 0.91, TLI = 0.89). The Cronbach’s α of the CAR Scale is 0.93, among which the Cronbach’s α of closeness, commitment and complementarity are 0.84, 0.82 and 0.84 respectively.

#### Thriving scale

The 10-item Thriving Scale compiled by Porath (2012) was used to measure athlete’ thriving [75]. The questionnaire was originally used to evaluate the thriving at work, but the author has adjusted it according to the sports background [61]. For example: “At work, I find myself learning often” → “In sports training and competition, I find myself learning often”; “At work, I feel alive and vital” → “In sports training and competition, I feel alive and vital”, etc. It consists of two dimensions: vitality and learning. The thriving scale has a good fit, CFA shows: χ^2^/df = 4.33, RMSEA = 0.10, SRMR = 0.07, CFI = 0.88, TLI = 0.85. The Cronbach’s α of the Thriving Scale is 0.85, among which the Cronbach’s α of vitality and learning are 0.76 and 0.74 respectively.

#### Athlete Engagement Questionnaire (AEQ)

The AEQ was compiled by Lonsdale (2007) and translated by Lv [11], the questionnaire has been used for many times in China, with χ^2^/df = 1.89, CFI = 0.95, TLI = 0.94, RMSEA = 0.06, SRMR = 0.04 in this study. There are 16 items, including four dimensions of self-confidence, dedication, vigor and enthusiasm. The Cronbach’s α of the AEQ is 0.96, among which the Cronbach’s α of self-confidence, dedication, vigor and enthusiasm are 0.92, 0.86, 0.89 and 0.91 respectively.

### Validity and reliability of the instrument

Each structure’s internal validity is evaluated using confirmatory factor analysis (CFA). CFA demonstrates how well the updated model fits the data: CMIN/DF = 2.29, RMSEA = 0.07,SRMR = 0.03, CFI = 0.98, TLI = 0.98. In Table 1, the composite reliability and internal consistency reliability are displayed. Each structure’s Cronbach’s α are greater than 0.7, indicating a satisfactory level of reliability [72]. Additionally, each structure’s CR score is higher than 0.8, demonstrating good all-around reliability [72]. There is an acceptable level of convergence effectiveness for each structure when the AVE exceeds 0.7 (Table 1) [72].

**Table 1:** Reliability and validity analysis.

Variables	Variables	Items	Cronbach’s α	CR	AVE	FL
Coach-Athlete Relationship	closeness	4	0.84	0.85	0.59	0.67–0.85
	commitment	3	0.82	0.84	0.64	0.68–0.86
	complementarity	4	0.84	0.80	0.57	0.69–0.83
Thriving	learning	5	0.74	0.80	0.46	0.35–0.79
	vitality	5	0.76	0.81	0.48	0.36–0.86
Athlete engagement	self-confidence	4	0.92	0.92	0.74	0.82–0.89
	vigour	4	0.89	0.89	0.68	0.76–0.86
	dedication	4	0.86	0.87	0.62	0.72–0.83
	enthusiasm	4	0.91	0.91	0.72	0.80–0.90

### Common Method Bias (CMB)

When collecting all structural data simultaneously using self-reporting surveys, CMB is a potential issue. This study used a balanced item order, anonymous questionnaire measurement, and standardized measurement in the questionnaire process to reduce the interference of CMB on validity. We discovered using Harman’s single factor test that the variance explained by the first principal component analysis factor was only 30.4%, which was less than the necessary threshold of 40%. According to CFA, the 9-factor model’s fit index was considerably higher than the single factor model’s fit index (χ^2^/df = 5.23, RMSEA = 0.12, SRMR = 0.09, CFI = 0.66, TLI = 0.64).

### Data analysis

SPSS 22.0 (IBM, Armonk, NY, USA) was used to input the questionnaire data for descriptive analysis, reliability analysis, and hierarchical regression analysis. Mplus 8.0 (Muthen & Muthen, Los Angeles, CA, USA) was used for the CMB test, CFA, and mediating effect model analysis.

## Results

### Descriptive analysis

A total of 358 questionnaires were distributed. After deleting the questionnaires with too short response time (less than 6 minutes) and consistent answers, 287 were effectively recovered. The effective rate was 80.1%. Among them, 185 cases were male (64.5%), and 102 cases were female (35.5%); 125 national second-level athletes (43.6%), 84 national first-level athletes (29.2%), 46 national elite athletes (16.1%), and 32 athletes lack sports information. The average age of athletes was 19.63 years (SD = 2.53), and the average training time was 6.78 years (SD = 3.85). Detail information in Table 2.

**Table 2:** Descriptive statistics of selected groups.

Variables	N	%
Total	287	100
Gender		
Male	185	35.5
Female	102	64.5
Age		
Under eighteen years old	54	18.8
Eighteen years old	20	7.0
Nineteen years old	44	15.3
Twenty years old	73	25.4
Twenty-one years old	36	12.5
Twenty-two years old	29	10.1
Twenty-three years old	13	4.5
Twenty-four years old	10	3.5
Over twenty-four years old	8	2.9
Sports Level		
National elite athletes	46	16.1
National first-level athletes	84	29.2
National second-level athletes	125	43.6
Lack spots information	32	11.1
Games		
Football	63	22.1
Basketball	54	18.8
Cricket	37	12.9
Volleyball	36	12.5
Ice hockey	20	6.9
Curling	15	5.2
Group aerobics	42	14.7
Others	20	6.9

### Correlational analysis

CAR, thriving, athlete engagement mean (M) and standard deviation (SD) are displayed in Table 3. The correlation coefficient between CAR, thriving, and athlete engagement is tested using Spearman’s analysis. According to the results, all variables are significant and correlated, with correlation coefficients ranging from 0.51 to 0.73, supporting the validity of the overall data of the measurement model.

**Table 3:** Descriptive statistics of model variables and correlations among model variables.

Component	M	SD	1	2	3
Coach-Athlete Relationship	4.54	0.48	—		
Thriving	4.15	0.56	0.51***	—	
Athlete Engagement	4.31	0.57	0.60***	0.73***	—

Note:

*** p < 0.001.

### Dominance analysis of different dimensions of CAR in predicting athlete engagement

After controlling for gender, age, years of training, and sports performance, it was found through regression analysis that CAR total points explained 36% of athlete engagement (*β* = 0.60, *t* = 12.6, *p* < 0.001). To understand the influence of three dimensions of CAR on athlete engagement, this study uses a multiple regression for dominance analysis. The dominance analysis method with model independence is used to calculate the change value of R^2^ after each explanatory variable is added to the sub-model without the variable itself to illustrate the explanatory effect of pertinent dimensions [75]. This method explains the relative contribution of each CAR dimension to the athlete engagement effect. Table 4 indicates that significant positive correlations between CAR dimensions and athlete engagement. This result indicates that each variable data can support the establishment of multiple regression models in this study. Table 5 indicates the multiple regression analysis results and the value added contribution of three dimensions of CAR on athlete engagement. The regression analysis results of closeness, commitment, and complementarity on athlete engagement are all significant (*β* = 0.219, 0.337, 0.348). Thus, Hypothesis 1 is verified. In addition, the change in value of R^2^ indicates that closeness, commitment, and complementarity contribute 30.37%, 34.55%, and 35.08% each to the explained variation of athlete engagement. This result indicates that three dimensions of CAR have varying degrees of predictive effect on athlete engagement; among them, complementarity contributed the most to the explained variation.

**Table 4:** Means, standard deviations and correlation coefficients among closeness, commitment, complementarity, and athlete engagement.

Component	M	SD	1	2	3	4
Closeness	4.62	0.49	—			
Commitment	4.50	0.56	0.68***	—		
Complementarity	4.50	0.54	0.74***	0.80***	—	
Athlete Engagement	4.31	0.57	0.47***	0.58***	0.59***	—

Note:

*** p < 0.001.

**Table 5:** Relative contribution of closeness, commitment, and complementarity on predicting athlete engagement.

Multidimensional Dimension of Coach-athlete Relationship	R^2^	Value-Added Contribution		
		X1	X2	X3
—	—	0.219	0.337	0.348
X1	0.219	—	0.009	0.002
X2	0.337	0.128	—	0.033
X3	0.348	0.132	0.044	—
X1X2	0.347	—	—	0.035
X1X3	0.351	—	0.031	—
X2X3	0.381	0.000	—	—
X1X2X3	0.381	—	—	—
Relative importance analysis	—	0.116	0.132	0.134
Predicted variance percentage	—	30.37	34.55	35.08

Note: X1, X2, X3 indicate closeness, commitment, complementarity.

### Mediating effect of thriving in CAR and athlete engagement

A confirmatory factor analysis was conducted on CAR, thriving, and athlete engagement before conducting the path model test [76]. Following Kline (1998) [77], χ^2^/df is recommended to be less than 3, CFI and TLI are recommended to be greater than 0.90, and RMSEA is recommended to be less than 0.08 for the model to be considered acceptable. In the current study, the measured model data was found to fit well (χ^2^/df = 2.29, CFI = 0.98, TLI = 0.98, RMSEA = 0.07, SRMR = 0.03).

**Fig 1:**
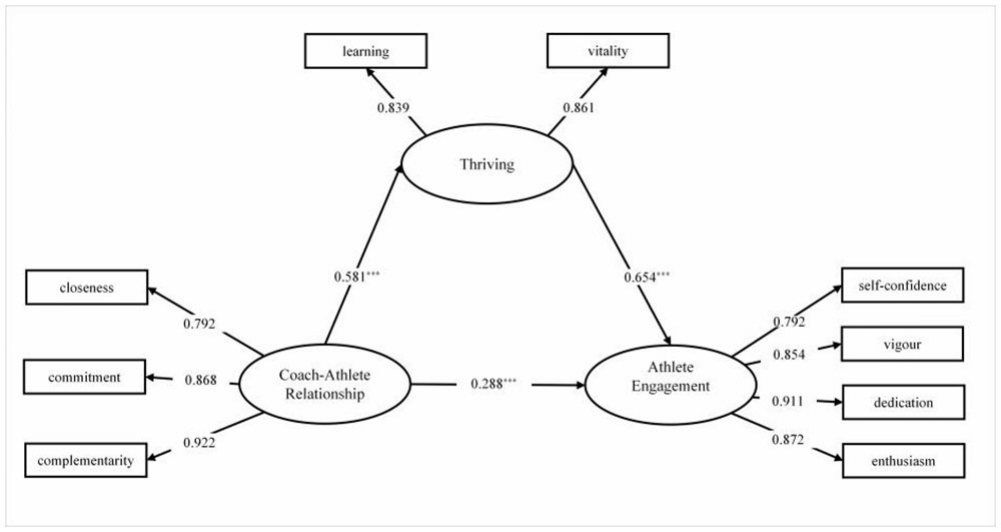
Mediating effect of thriving in CAR on athlete engagement. χ2/df = 2.29, RMSEA = 0.07, SRMR = 0.03,CFI = 0.98, TLI = 0.98.

In the mediating effect model (Fig 1 and Table 6), all paths are significant (*p* < 0.001), which indicates that the mediating effect of thriving is significant. In addition, the load of each observation index is 0.779~0.911, indicating that the reliability of each observation variable is reliable. Deviation corrected bootstrap test was also used to test the model and 5000 samples were repeated to calculate the 95% confidence interval [78]. Results of the final model show that the confidence interval of the mediating effect from the CAR to the athlete engagement is [0.305, 0.486] and does not include zero. This indicates that the mediating effect is, again, significant. Table 6 also shows that the direct effect of the CAR on athlete engagement is 0.288 and the mediating effect (indirect effect) is 0.380, therein accounting for 56.9% of the total effect. Thriving therefore plays a partial mediating role in the CAR and the athlete engagement, ultimately verifying H2.

**Table 6:** Effect analysis of latent variables.

Influence Path	Standardized Effect Value	Significance	%
Coach-Athlete Relationship→Athlete Engagement	0.288	***	43.1
Coach-Athlete Relationship→Thriving→Athlete Engagement	0.581 × 0.654 = 0.380	***	56.9
The total effect	0.288+ 0.380 = 0.668	***	——

Note:

*** p < 0.001.

## Discussion

### Direct effect of CAR on athlete engagement

The influence of CAR on athlete engagement reflects a practical application of interpersonal dynamics in competitive performance to a certain extent. Some problems in competitive sports, such as coach-athlete conflict, parental over-involvement, lack of support, depression, loneliness, withdrawal intention, aggressiveness, and power struggle, are fundamentally interpersonal [32]. The results suggest that high-quality CAR indicates that coaches and athletes maintain an efficient interactive mode, wherein athletes receive sufficient knowledge, guidance and social, emotional, and informational support from their coaches. These factors significantly enhance athletes’ sense of security, belonging, and drive, thereby helping them participate in training and competition with full enthusiasm and vitality [7,11,34,79]. The 3Cs theory embodies dual subjectivity and covers the three dimensions of knowledge, emotion, and meaning. If coaches and athletes maintain long-term cooperation intentions, respect and emotional trust, and actively engage in interactive behavior, then athletes can participate well in training and competition, bringing a positive attitude, increased focus, and self-motivation in adversity.

The dominance analysis shows that the three dimensions of CAR significantly and positively predict athlete engagement, with complementarity having a more important effect on athlete engagement. Previous studies have indicated that factors such as mutual trust, respect, belief, support, cooperation, communication, and understanding are considered the most important relationship components that contribute to performance success and satisfaction [32]. Conversely, lack of trust, lack of respect, excessive dominance, and blind obedience, and verbal, physical, and sexual exploitation are considered components that undermine coaches’ and athletes’ welfare [32]. Complementarity reflects the behavior pattern of coaches and athletes working together to improve performance. Mutual support and understanding in goals, abilities, and personal relationships contribute to the formation of an encouraging atmosphere, thereby stimulating athletes’ autonomous motivation and promoting confidence and dedication in competition and training.

Moreover, commitment accounted for 34.55% of the variance in interpreting CAR’s influence on athlete engagement. Commitment represents the willingness of coaches and athletes to establish and maintain cooperative relationships, thereby reflecting their psychological tendency to unite for common interests. Athletes with a high sense of commitment also possess the courage to express their own needs and personal values, assisting coaches and athletes in forming an equal, voluntary, inclusive, and progressive community of interests [34]. According to strong culture theory, organizational commitment arises from the organizational atmosphere and motivation. When the team and its members share consistent goals and receive the internal recognition from the organization, members experience increased job satisfaction and work engagement [80]. Therefore, in CAR management, setting common goal and emphasizing mutual cooperation, trust, and support encourage coaches and athletes to exhibit higher commitment and complementarity to each other, ultimately improving athletes’ engagement behavior.

Finally, closeness contributed only 30.37% of the variance in interpreting CAR’s influence on athlete engagement. Closeness reflects the emotional attachment between coaches and athletes. Some studies have argued that closeness plays the most important role in athletes’ social support of athletes [34]. Therefore, emphasizing closeness is likely to have an important role in the engagement of athletes within younger age ranges and with stronger psychological dependence.

### Mediating effect of thriving between CAR and athlete engagement

The research results indicate that the mediating effect of thriving between CAR and athlete engagement is greater than the direct effect of CAR on athlete engagement. Thus, thriving is an important embodiment of good CAR, and their interaction influences the development of athlete engagement. According to the elite athlete thriving model, athlete thriving results from the interaction of personality and environment. The satisfaction of psychological needs stimulates internal motivation, thereby promoting athletes’ propensity to thrive and further enhancing their sports performance, self-confidence, motivation, and individual healthy development [62]. Therefore, thriving fulfills autonomy needs between CAR and athlete engagement and represents a form of self-cognition of good mental health. These positive experiences inspire athletes to gain confidence, dedication, enthusiasm, and vitality.

Extant literature predominantly bases the thriving interpretation framework on the needs satisfaction theory [62,81,82], given that thriving is defined as individuals’ sense of vitality and progress in the process of self-development. Growth, integrity, and wellbeing depend on the satisfaction of their needs, and providing observable and meaningful positive consequences for thriving. In Jowett’s CAR theory [31,33,73], closeness, commitment, and complementarity correspond to the emotional, cognitive and behavioral characteristics of the CAR structure, respectively. All three also correspond well to the needs of relatedness, autonomy, and competence according to the needs satisfaction theory [18,67]. Therefore, when a good CAR is available, athletes can meet their own needs well. These conditions further stimulate athletes’ autonomous motivation, thereby promoting greater engagement in training and competition.

According to Spreitzer’s thriving model (2012), thriving is the shared experience of vitality and learning. Although each dimension represents some progress in personal development, they can only strengthen each other and form a thriving experience when coordinated [75]. For example, if athletes have a need for progress but feels that training and interpersonal activities have drained their energy, their thriving decreases. Conversely, if athletes feel energetic in training and competition but lack the need for progress, they experience subjective wellbeing and energy rather than actual thriving. Therefore, in daily CAR management, the simultaneous satisfaction and development of athletes’ relatedness, autonomy, and competence needs must be given attention. Only when these conditions are met can the sense of thriving be thoroughly enhanced, which can be achieved through the cultivation of closeness, commitment, and complementarity.

In collectivist cultures, coaches and athletes tend to prioritize maintaining interpersonal relationships and attribute significant value to interdependence, because collectivist cultures emphasize people’s interdependence, social embeddedness, and obligations and loyalty to their internal groups [16,34,83,84]. This cultural background positively influences the relationship between CAR and athlete engagement [34]. When CAR is in a harmonious state, athletes can focus on training or competition to avoid losses caused by interpersonal pressure, thereby maintaining sports vitality. At the same time, coaches who have a close relationship with athletes provide guidance based on athletes’ needs, personality, and athletic ability, helping them progress in training and competition. When athletes simultaneously experience a sense of vitality and learning, they will enter a thriving state. Athletes in this state are not content with maintaining the status quo and often have the growth demands centered on development, progress, and breakthrough. Driven by these growth demands, these athletes exhibit athletic engagement in training and competition.

## Conclusion

These two hypotheses are both supported by the results of this study. Athlete engagement is significantly and positively predicted by CAR and all dimensions, in which complementarity plays an important role. The CAR and athlete engagement were also partially mediated by Thriving. Among the total effects, this mediating effect accounted for 56.9%. The mediating effect model developed also fits well, providing some justification for how CAR affects athlete engagement.

The CAR on athlete engagement reflects a practical application of interpersonal dynamics in competitive sports to a certain extent. To improve athlete engagement, the following points can be considered. First, by setting common goals and emphasizing mutual cooperation, trust and support, coaches and athletes can develop a high sense of commitment and complementarity to each other, which, in turn, enhances athlete engagement. Second, addressing athletes’ vitality and progress needs effectively harnesses CAR resources to promote athlete engagement, which can be achieved through the cultivation of closeness, commitment, and complementarity. Third, sports teams should prioritize fostering athletes’ ability to thrive and create a dynamic, positive atmosphere within the team to ensure their athletic performance and mental health. Future research can track and compare the influence of CAR improvement on thriving and athlete engagement from coaches and athletes’ perspectives.

### Implications

The effect of CAR on athlete engagement reflects a practical application of interpersonal dynamics in competitive sports to a certain extent. First, a good CAR acts as a driving force for generating athlete engagement, reflecting the dynamic process of coaches and athletes uniting to achieve their goals and produce positive experiences. Second, this study highlights that athletes’ thriving is an important manifestation of a good CAR. In sports practice, promoting athlete thriving can be achieved by establishing a good CAR, which can be predicted by questionnaires or by observing athletes’ initiative and vitality. Third, the 3Cs theory of CAR greatly coincides with athletes’ relatedness, autonomy, and competence needs. Thus, daily CAR management can improve athletes’ thriving and engagement through the cultivation of closeness, commitment, and complementarity. For example, incorporating CAR management into coaches and athletes’ training can involve topics such as gratitude and forgiveness, interpersonal communication, and conflict management. In addition, goal-setting training can enhance commitment in CAR, thereby promoting harmony and efficiency in the environmental, cognitive, and emotional aspects of CAR.

Although thriving and athlete engagement share similarities in content, both reflecting athletes’ positive states in sports [1,3,5,59,62] thriving focuses on the perception of a state, with its motivation tendency and subsequent emotions [1]. Conversely, athlete engagement is concerned with individuals’ cognition in sports behavior, emphasizing coping styles in specific situations [3]. Notable differences exist between them during their occurrence and the psychological objects they describe. Thus, effectively addressing athletes’ vitality and progress needs mobilizes CAR to promote athlete engagement.

### Limitations and future study

First, although cross-sectional studies provide valuable information, they cannot determine causality, and this study does not consider potential adverse effects. Future studies could employ longitudinal tracking and experimental designs to test these findings. Second, the research sample relies solely on athletes’ perspectives, which may limit the authenticity of CAR. Future studies could incorporate coaches’ perspectives to verify these results. Third, this study overlooks the effects of different sports characteristics and levels on the findings. Future research could explore sports, team size, leader and member roles, team achievements, and cultural backgrounds to enhance the findings. Fourth, the results are based on a sample of Chinese athletes, so future studies in other contexts are recommended to improve the generalizability of the results. Fifth, the data collection for this study occurred during the COVID-19 pandemic. Although China did not experience a large-scale outbreak at the time, the potential impact of the pandemic on the findings cannot be dismissed. Some studies have suggested that training suspensions due to the pandemic can cause emotional interference and motivation-forming dysfunction in athletes [85]. Future research could further explore the relationship among CAR, thriving, and athlete engagement, considering the impact of the pandemic on training behavior.

## Supporting information

S1 File Calculation process of reliability and validity analysis. (DOCX)

S2 File Calculation process of descriptive statistics and correlations among model variables. (DOCX)

S3 File Calculation process of CAR constructs predicting relative contribution of athlete engagement. (DOCX)

S4 File Calculation process of direct and indirect effect. (DOCX)

S5 File Calculation process of mediating role of thriving in CAR on athlete engagement. (DOCX)

S6 File Raw data. (XLSX)

S7 File The questionnaire used in this study. (DOCX)
