# Supplementary material for: Association between coach-athlete relationship and athlete engagement in Chinese team sports: The mediating effect of thriving
Source: PLoS One. 2023 Aug 17;18(8):e0289979. doi: 10.1371/journal.pone.0289979 (PMC10434943; doi:10.1371/journal.pone.0289979)
Supplement: S1 File — (DOCX) [file pone.0289979.s001.docx]

*Athlete Engagement Questionnaire (AEQ).*

**The Cronbach's a of the AEQ is 0.96, among which the Cronbach's a of self-confidence, dedication, vigor and enthusiasm are 0.92, 0.89, 0.77 and 0.91 respectively.**

**
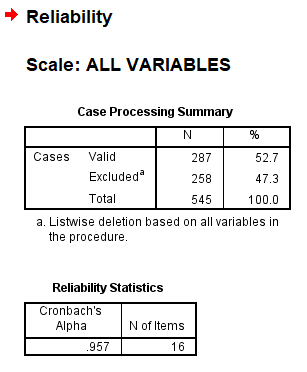
**

**
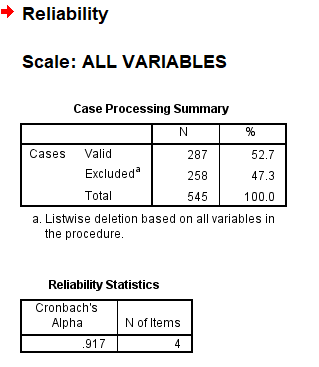
**

**
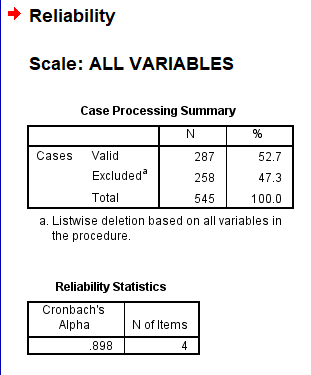
**

**
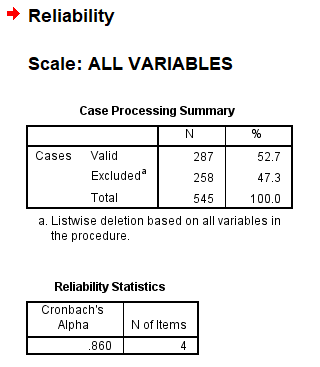
**

**
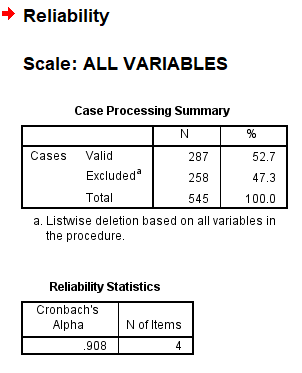
**

**The AEQ was compiled by Lonsdale (2007) and translated by Lv [11], the questionnaire has been used for many times in China, with χ2/df = 1.89, CFI = 0.95, TLI = 0.94, RMSEA = 0.06, SRMR = 0.04 in this study.**

**
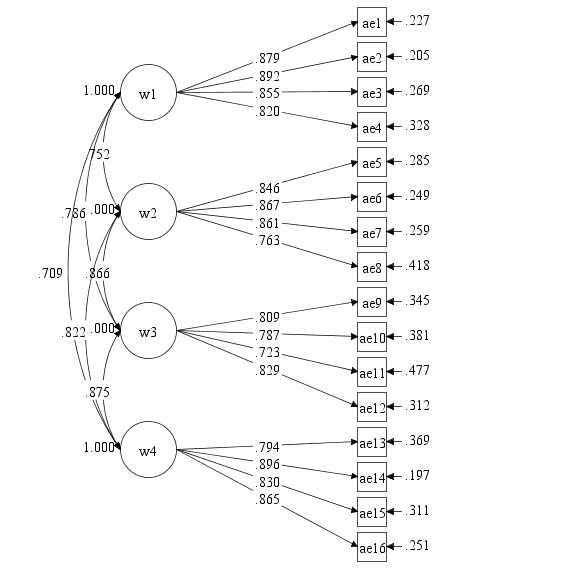
**

**Mplus VERSION 8.3**

**MUTHEN & MUTHEN**

**04/11/2023 5:44 PM**

**INPUT INSTRUCTIONS**

**DATA:**

**FILE IS C:\Users\DELL\Desktop\shu\+AT4+CAR3\3添加CAR3-1.dat;**

**VARIABLE:**

**NAMES ARE xh gender ag syd ds grade ydxm ydnx cdnx AT1 AT2 AT3**

**AT4 AT5 ATw1 ATw1A AT6 AT7 AT8 AT9 AT10 ATw2 ATw2A ATsum**

**ATsumA CAR1 CAR5 CAR9 CAR11 CARw1 CARw1A CAR2 CAR6 CAR7**

**CARw2 CARw2A CAR3 CAR4 CAR8 CAR10 CARw3 CARw3A CARsum**

**CARsumA AE1 AE2 AE3 AE4 AEw1 AEw1A AE5 AE6 AE7 AE8 AEw2**

**AEw2A AE9 AE10 AE11 AE12 AEw3 AEw3A AE13 AE14 AE15 AE16**

**AEw4 AEw4A AEsum AEsumA;**

**USEVARIABLES ARE**

**AE1 AE2 AE3 AE4 AE5 AE6 AE7 AE8 AE9 AE10 AE11 AE12 AE13 AE14 AE15 AE16;**

**ANALYSIS:**

**TYPE IS GENERAL;**

**ESTIMATOR IS MLM;**

**MODEL:**

**W1 BY AE1 AE2 AE3 AE4;**

**W2 BY AE5 AE6 AE7 AE8;**

**W3 BY AE9 AE10 AE11 AE12;**

**W4 BY AE13 AE14 AE15 AE16;**

**OUTPUT:**

**SAMPSTAT STDYX MODINDICES;**

**INPUT READING TERMINATED NORMALLY**

**SUMMARY OF ANALYSIS**

**Number of groups 1**

**Number of observations 287**

**Number of dependent variables 16**

**Number of independent variables 0**

**Number of continuous latent variables 4**

**Observed dependent variables**

**Continuous**

**AE1 AE2 AE3 AE4 AE5 AE6**

**AE7 AE8 AE9 AE10 AE11 AE12**

**AE13 AE14 AE15 AE16**

**Continuous latent variables**

**W1 W2 W3 W4**

**Estimator MLM**

**Information matrix EXPECTED**

**Maximum number of iterations 1000**

**Convergence criterion 0.500D-04**

**Maximum number of steepest descent iterations 20**

**Input data file(s)**

**C:\Users\DELL\Desktop\shu\+AT4+CAR3\3添加CAR3-1.dat**

**Input data format FREE**

**SAMPLE STATISTICS**

**SAMPLE STATISTICS**

**Means**

**AE1 AE2 AE3 AE4 AE5**

**________ ________ ________ ________ ________**

**4.251 4.293 4.258 4.213 4.226**

**Means**

**AE6 AE7 AE8 AE9 AE10**

**________ ________ ________ ________ ________**

**4.220 4.247 4.164 4.373 4.334**

**Means**

**AE11 AE12 AE13 AE14 AE15**

**________ ________ ________ ________ ________**

**4.394 4.460 4.380 4.362 4.321**

**Means**

**AE16**

**________**

**4.418**

**Covariances**

**AE1 AE2 AE3 AE4 AE5**

**________ ________ ________ ________ ________**

**AE1 0.529**

**AE2 0.414 0.486**

**AE3 0.378 0.364 0.484**

**AE4 0.389 0.398 0.402 0.627**

**AE5 0.323 0.303 0.311 0.377 0.579**

**AE6 0.321 0.281 0.281 0.378 0.424**

**AE7 0.279 0.283 0.281 0.331 0.432**

**AE8 0.290 0.259 0.282 0.355 0.353**

**AE9 0.269 0.239 0.224 0.304 0.299**

**AE10 0.306 0.257 0.262 0.305 0.287**

**AE11 0.243 0.230 0.250 0.289 0.301**

**AE12 0.240 0.242 0.244 0.261 0.290**

**AE13 0.257 0.262 0.285 0.289 0.322**

**AE14 0.289 0.291 0.262 0.327 0.343**

**AE15 0.292 0.276 0.280 0.312 0.325**

**AE16 0.257 0.250 0.262 0.308 0.334**

**Covariances**

**AE6 AE7 AE8 AE9 AE10**

**________ ________ ________ ________ ________**

**AE6 0.576**

**AE7 0.427 0.569**

**AE8 0.410 0.416 0.660**

**AE9 0.298 0.277 0.294 0.394**

**AE10 0.317 0.318 0.367 0.318 0.522**

**AE11 0.321 0.261 0.301 0.230 0.262**

**AE12 0.275 0.266 0.245 0.264 0.285**

**AE13 0.310 0.331 0.321 0.252 0.312**

**AE14 0.346 0.346 0.394 0.273 0.335**

**AE15 0.337 0.328 0.345 0.250 0.290**

**AE16 0.333 0.290 0.336 0.245 0.268**

**Covariances**

**AE11 AE12 AE13 AE14 AE15**

**________ ________ ________ ________ ________**

**AE11 0.490**

**AE12 0.258 0.374**

**AE13 0.324 0.320 0.514**

**AE14 0.338 0.293 0.382 0.573**

**AE15 0.292 0.274 0.370 0.438 0.601**

**AE16 0.306 0.264 0.308 0.413 0.396**

**Covariances**

**AE16**

**________**

**AE16 0.473**

**Correlations**

**AE1 AE2 AE3 AE4 AE5**

**________ ________ ________ ________ ________**

**AE1 1.000**

**AE2 0.817 1.000**

**AE3 0.746 0.750 1.000**

**AE4 0.675 0.720 0.729 1.000**

**AE5 0.583 0.571 0.587 0.625 1.000**

**AE6 0.582 0.531 0.533 0.630 0.735**

**AE7 0.509 0.538 0.536 0.553 0.752**

**AE8 0.491 0.457 0.499 0.553 0.571**

**AE9 0.589 0.547 0.514 0.611 0.625**

**AE10 0.583 0.511 0.521 0.533 0.521**

**AE11 0.477 0.471 0.514 0.522 0.565**

**AE12 0.540 0.567 0.573 0.539 0.622**

**AE13 0.492 0.524 0.572 0.508 0.589**

**AE14 0.525 0.552 0.498 0.546 0.596**

**AE15 0.518 0.510 0.519 0.508 0.550**

**AE16 0.514 0.522 0.546 0.566 0.638**

**Correlations**

**AE6 AE7 AE8 AE9 AE10**

**________ ________ ________ ________ ________**

**AE6 1.000**

**AE7 0.745 1.000**

**AE8 0.666 0.679 1.000**

**AE9 0.626 0.585 0.577 1.000**

**AE10 0.578 0.583 0.625 0.700 1.000**

**AE11 0.605 0.495 0.530 0.523 0.518**

**AE12 0.594 0.577 0.494 0.688 0.645**

**AE13 0.570 0.612 0.551 0.560 0.602**

**AE14 0.602 0.606 0.641 0.574 0.613**

**AE15 0.573 0.561 0.547 0.513 0.518**

**AE16 0.639 0.559 0.601 0.567 0.539**

**Correlations**

**AE11 AE12 AE13 AE14 AE15**

**________ ________ ________ ________ ________**

**AE11 1.000**

**AE12 0.603 1.000**

**AE13 0.646 0.730 1.000**

**AE14 0.639 0.634 0.703 1.000**

**AE15 0.538 0.578 0.665 0.746 1.000**

**AE16 0.635 0.628 0.625 0.793 0.742**

**Correlations**

**AE16**

**________**

**AE16 1.000**

**UNIVARIATE SAMPLE STATISTICS**

**UNIVARIATE HIGHER-ORDER MOMENT DESCRIPTIVE STATISTICS**

**Variable/ Mean/ Skewness/ Minimum/ % with Percentiles**

**Sample Size Variance Kurtosis Maximum Min/Max 20%/60% 40%/80% Median**

**AE1 4.251 -0.695 2.000 1.74% 4.000 4.000 4.000**

**287.000 0.529 0.091 5.000 40.42% 5.000 5.000**

**AE2 4.293 -0.963 1.000 0.35% 4.000 4.000 4.000**

**287.000 0.486 1.723 5.000 40.77% 5.000 5.000**

**AE3 4.258 -0.583 2.000 1.05% 4.000 4.000 4.000**

**287.000 0.484 -0.093 5.000 39.37% 4.000 5.000**

**AE4 4.213 -0.775 2.000 3.14% 4.000 4.000 4.000**

**287.000 0.627 0.060 5.000 41.11% 5.000 5.000**

**AE5 4.226 -0.737 1.000 0.35% 4.000 4.000 4.000**

**287.000 0.579 0.338 5.000 40.77% 5.000 5.000**

**AE6 4.220 -0.773 1.000 0.35% 4.000 4.000 4.000**

**287.000 0.576 0.530 5.000 39.72% 4.000 5.000**

**AE7 4.247 -0.978 1.000 0.70% 4.000 4.000 4.000**

**287.000 0.569 1.505 5.000 40.77% 5.000 5.000**

**AE8 4.164 -0.854 1.000 0.70% 4.000 4.000 4.000**

**287.000 0.660 0.751 5.000 38.33% 4.000 5.000**

**AE9 4.373 -0.653 2.000 0.70% 4.000 4.000 4.000**

**287.000 0.394 0.282 5.000 44.60% 5.000 5.000**

**AE10 4.334 -1.100 1.000 0.35% 4.000 4.000 4.000**

**287.000 0.522 1.716 5.000 45.64% 5.000 5.000**

**AE11 4.394 -1.144 1.000 0.35% 4.000 4.000 4.000**

**287.000 0.490 1.834 5.000 49.83% 5.000 5.000**

**AE12 4.460 -0.853 2.000 0.70% 4.000 4.000 5.000**

**287.000 0.374 0.639 5.000 51.57% 5.000 5.000**

**AE13 4.380 -1.048 2.000 2.09% 4.000 4.000 4.000**

**287.000 0.514 0.896 5.000 49.83% 5.000 5.000**

**AE14 4.362 -1.386 1.000 0.70% 4.000 4.000 4.000**

**287.000 0.573 2.669 5.000 49.13% 5.000 5.000**

**AE15 4.321 -1.162 1.000 0.70% 4.000 4.000 4.000**

**287.000 0.601 1.666 5.000 47.74% 5.000 5.000**

**AE16 4.418 -1.213 1.000 0.35% 4.000 4.000 5.000**

**287.000 0.473 2.170 5.000 51.22% 5.000 5.000**

**THE MODEL ESTIMATION TERMINATED NORMALLY**

**MODEL FIT INFORMATION**

**Number of Free Parameters 54**

**Loglikelihood**

**H0 Value -3190.068**

**H1 Value -3006.328**

**Information Criteria**

**Akaike (AIC) 6488.135**

**Bayesian (BIC) 6685.747**

**Sample-Size Adjusted BIC 6514.507**

**(n* = (n + 2) / 24)**

**Chi-Square Test of Model Fit**

**Value 167.043***

**Degrees of Freedom 98**

**P-Value 0.0000**

**Scaling Correction Factor 2.1999**

**for MLM**

*** The chi-square value for MLM, MLMV, MLR, ULSMV, WLSM and WLSMV cannot be used**

**for chi-square difference testing in the regular way. MLM, MLR and WLSM**

**chi-square difference testing is described on the Mplus website. MLMV, WLSMV,**

**and ULSMV difference testing is done using the DIFFTEST option.**

**RMSEA (Root Mean Square Error Of Approximation)**

**Estimate 0.050**

**90 Percent C.I. 0.036 0.062**

**Probability RMSEA <= .05 0.507**

**CFI/TLI**

**CFI 0.961**

**TLI 0.953**

**Chi-Square Test of Model Fit for the Baseline Model**

**Value 1903.383**

**Degrees of Freedom 120**

**P-Value 0.0000**

**SRMR (Standardized Root Mean Square Residual)**

**Value 0.041**

**MODEL RESULTS**

**Two-Tailed**

**Estimate S.E. Est./S.E. P-Value**

**W1 BY**

**AE1 1.000 0.000 999.000 999.000**

**AE2 0.971 0.060 16.124 0.000**

**AE3 0.930 0.071 13.175 0.000**

**AE4 1.015 0.074 13.630 0.000**

**W2 BY**

**AE5 1.000 0.000 999.000 999.000**

**AE6 1.022 0.067 15.181 0.000**

**AE7 1.009 0.066 15.207 0.000**

**AE8 0.963 0.084 11.529 0.000**

**W3 BY**

**AE9 1.000 0.000 999.000 999.000**

**AE10 1.119 0.079 14.201 0.000**

**AE11 0.996 0.107 9.305 0.000**

**AE12 0.998 0.066 15.099 0.000**

**W4 BY**

**AE13 1.000 0.000 999.000 999.000**

**AE14 1.190 0.123 9.686 0.000**

**AE15 1.130 0.122 9.278 0.000**

**AE16 1.045 0.121 8.667 0.000**

**W2 WITH**

**W1 0.310 0.031 9.890 0.000**

**W3 WITH**

**W1 0.256 0.029 8.798 0.000**

**W2 0.283 0.024 11.737 0.000**

**W4 WITH**

**W1 0.258 0.033 7.885 0.000**

**W2 0.301 0.036 8.265 0.000**

**W3 0.253 0.029 8.860 0.000**

**Intercepts**

**AE1 4.251 0.043 98.975 0.000**

**AE2 4.293 0.041 104.341 0.000**

**AE3 4.258 0.041 103.679 0.000**

**AE4 4.213 0.047 90.105 0.000**

**AE5 4.226 0.045 94.068 0.000**

**AE6 4.220 0.045 94.227 0.000**

**AE7 4.247 0.045 95.352 0.000**

**AE8 4.164 0.048 86.854 0.000**

**AE9 4.373 0.037 118.004 0.000**

**AE10 4.334 0.043 101.610 0.000**

**AE11 4.394 0.041 106.381 0.000**

**AE12 4.460 0.036 123.575 0.000**

**AE13 4.380 0.042 103.464 0.000**

**AE14 4.362 0.045 97.671 0.000**

**AE15 4.321 0.046 94.410 0.000**

**AE16 4.418 0.041 108.800 0.000**

**Variances**

**W1 0.409 0.046 8.879 0.000**

**W2 0.414 0.048 8.620 0.000**

**W3 0.258 0.031 8.227 0.000**

**W4 0.324 0.055 5.923 0.000**

**Residual Variances**

**AE1 0.120 0.017 6.882 0.000**

**AE2 0.100 0.015 6.841 0.000**

**AE3 0.130 0.021 6.294 0.000**

**AE4 0.206 0.023 8.870 0.000**

**AE5 0.165 0.019 8.773 0.000**

**AE6 0.143 0.024 5.909 0.000**

**AE7 0.147 0.018 8.400 0.000**

**AE8 0.276 0.030 9.118 0.000**

**AE9 0.136 0.017 7.788 0.000**

**AE10 0.199 0.027 7.274 0.000**

**AE11 0.233 0.033 7.080 0.000**

**AE12 0.117 0.015 7.586 0.000**

**AE13 0.190 0.026 7.427 0.000**

**AE14 0.113 0.018 6.259 0.000**

**AE15 0.187 0.058 3.225 0.001**

**AE16 0.119 0.016 7.341 0.000**

**STANDARDIZED MODEL RESULTS**

**STDYX Standardization**

**Two-Tailed**

**Estimate S.E. Est./S.E. P-Value**

**W1 BY**

**AE1 0.879 0.021 42.517 0.000**

**AE2 0.892 0.015 58.415 0.000**

**AE3 0.855 0.024 36.080 0.000**

**AE4 0.820 0.024 34.533 0.000**

**W2 BY**

**AE5 0.846 0.020 43.310 0.000**

**AE6 0.867 0.024 36.229 0.000**

**AE7 0.861 0.021 40.516 0.000**

**AE8 0.763 0.033 23.148 0.000**

**W3 BY**

**AE9 0.809 0.027 29.515 0.000**

**AE10 0.787 0.027 28.786 0.000**

**AE11 0.723 0.040 18.183 0.000**

**AE12 0.829 0.026 32.128 0.000**

**W4 BY**

**AE13 0.794 0.036 21.864 0.000**

**AE14 0.896 0.019 46.003 0.000**

**AE15 0.830 0.048 17.311 0.000**

**AE16 0.865 0.021 40.927 0.000**

**W2 WITH**

**W1 0.752 0.039 19.163 0.000**

**W3 WITH**

**W1 0.786 0.036 21.819 0.000**

**W2 0.866 0.029 30.094 0.000**

**W4 WITH**

**W1 0.709 0.047 15.074 0.000**

**W2 0.822 0.029 28.311 0.000**

**W3 0.875 0.025 34.855 0.000**

**Intercepts**

**AE1 5.842 0.276 21.148 0.000**

**AE2 6.159 0.373 16.503 0.000**

**AE3 6.120 0.281 21.780 0.000**

**AE4 5.319 0.254 20.976 0.000**

**AE5 5.553 0.279 19.900 0.000**

**AE6 5.562 0.286 19.424 0.000**

**AE7 5.628 0.330 17.033 0.000**

**AE8 5.127 0.281 18.247 0.000**

**AE9 6.966 0.312 22.357 0.000**

**AE10 5.998 0.351 17.088 0.000**

**AE11 6.279 0.324 19.382 0.000**

**AE12 7.294 0.380 19.221 0.000**

**AE13 6.107 0.348 17.571 0.000**

**AE14 5.765 0.365 15.779 0.000**

**AE15 5.573 0.356 15.633 0.000**

**AE16 6.422 0.363 17.674 0.000**

**Variances**

**W1 1.000 0.000 999.000 999.000**

**W2 1.000 0.000 999.000 999.000**

**W3 1.000 0.000 999.000 999.000**

**W4 1.000 0.000 999.000 999.000**

**Residual Variances**

**AE1 0.227 0.036 6.239 0.000**

**AE2 0.205 0.027 7.537 0.000**

**AE3 0.269 0.041 6.649 0.000**

**AE4 0.328 0.039 8.438 0.000**

**AE5 0.285 0.033 8.636 0.000**

**AE6 0.249 0.041 5.999 0.000**

**AE7 0.259 0.037 7.074 0.000**

**AE8 0.418 0.050 8.310 0.000**

**AE9 0.345 0.044 7.779 0.000**

**AE10 0.381 0.043 8.848 0.000**

**AE11 0.477 0.058 8.279 0.000**

**AE12 0.312 0.043 7.284 0.000**

**AE13 0.369 0.058 6.395 0.000**

**AE14 0.197 0.035 5.643 0.000**

**AE15 0.311 0.080 3.899 0.000**

**AE16 0.251 0.037 6.862 0.000**

**R-SQUARE**

**Observed Two-Tailed**

**Variable Estimate S.E. Est./S.E. P-Value**

**AE1 0.773 0.036 21.259 0.000**

**AE2 0.795 0.027 29.207 0.000**

**AE3 0.731 0.041 18.040 0.000**

**AE4 0.672 0.039 17.267 0.000**

**AE5 0.715 0.033 21.655 0.000**

**AE6 0.751 0.041 18.114 0.000**

**AE7 0.741 0.037 20.258 0.000**

**AE8 0.582 0.050 11.574 0.000**

**AE9 0.655 0.044 14.757 0.000**

**AE10 0.619 0.043 14.393 0.000**

**AE11 0.523 0.058 9.092 0.000**

**AE12 0.688 0.043 16.064 0.000**

**AE13 0.631 0.058 10.932 0.000**

**AE14 0.803 0.035 23.002 0.000**

**AE15 0.689 0.080 8.656 0.000**

**AE16 0.749 0.037 20.463 0.000**

**QUALITY OF NUMERICAL RESULTS**

**Condition Number for the Information Matrix 0.170E-02**

**(ratio of smallest to largest eigenvalue)**

**MODEL MODIFICATION INDICES**

**NOTE: Modification indices for direct effects of observed dependent variables**

**regressed on covariates may not be included. To include these, request**

**MODINDICES (ALL).**

**Minimum M.I. value for printing the modification index 10.000**

**M.I. E.P.C. Std E.P.C. StdYX E.P.C.**

**BY Statements**

**W3 BY AE13 15.378 0.938 0.477 0.665**

**W4 BY AE11 11.245 0.786 0.448 0.640**

**WITH Statements**

**AE13 WITH AE12 13.086 0.056 0.056 0.375**

**DIAGRAM INFORMATION**

**Use View Diagram under the Diagram menu in the Mplus Editor to view the diagram.**

**If running Mplus from the Mplus Diagrammer, the diagram opens automatically.**

**Diagram output**

**c:\users\dell\desktop\数据\原始数据\mptext7.dgm**

**Beginning Time: 17:44:54**

**Ending Time: 17:44:55**

**Elapsed Time: 00:00:01**

**MUTHEN & MUTHEN**

**3463 Stoner Ave.**

**Los Angeles, CA 90066**

**Tel: (310) 391-9971**

**Fax: (310) 391-8971**

**Web: www.StatModel.com**

**Support: Support@StatModel.com**

**Copyright (c) 1998-2019 Muthen & Muthen**

*Thriving Scale.*

**Thriving Scale is 0.85, among which the Cronbach's a of learning and vitality are 0.74 and 0.76 respectively.**


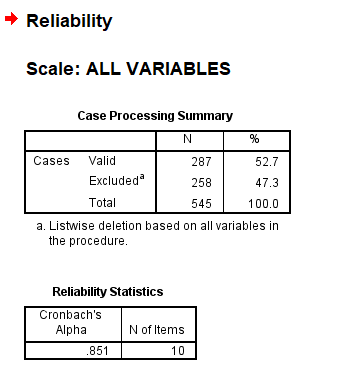


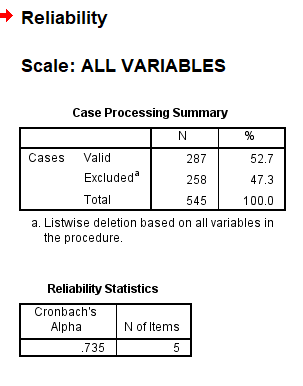


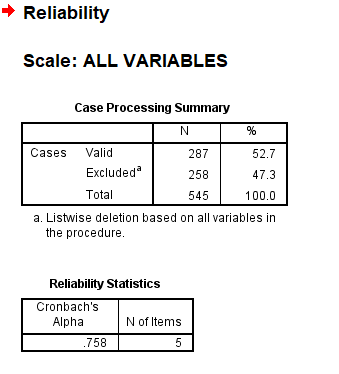


**The thriving scale has a good fit, CFA shows: χ^2^/df = 4.33, RMSEA = 0.10, SRMR = 0.07, CFI = 0.88, TLI = 0.85.**


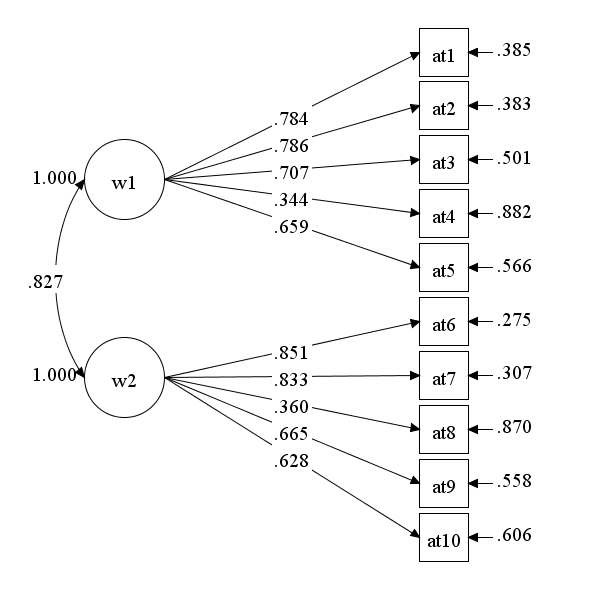


Mplus VERSION 8.3

MUTHEN & MUTHEN

04/11/2023 2:58 PM

INPUT INSTRUCTIONS

DATA:

FILE IS C:\Users\DELL\Desktop\shu\+AT4+CAR3\3 CAR3-1.dat;

VARIABLE:

NAMES ARE xh gender ag syd ds grade ydxm ydnx cdnx AT1 AT2 AT3

AT4 AT5 ATw1 ATw1A AT6 AT7 AT8 AT9 AT10 ATw2 ATw2A ATsum

ATsumA CAR1 CAR5 CAR9 CAR11 CARw1 CARw1A CAR2 CAR6 CAR7

CARw2 CARw2A CAR3 CAR4 CAR8 CAR10 CARw3 CARw3A CARsum

CARsumA AE1 AE2 AE3 AE4 AEw1 AEw1A AE5 AE6 AE7 AE8 AEw2

AEw2A AE9 AE10 AE11 AE12 AEw3 AEw3A AE13 AE14 AE15 AE16

AEw4 AEw4A AEsum AEsumA;

USEVARIABLES ARE

AT1 AT2 AT3 AT4 AT5 AT6 AT7 AT8 AT9 AT10;

ANALYSIS:

TYPE IS GENERAL;

ESTIMATOR IS MLM;

MODEL:

W1 BY AT1 AT2 AT3 AT4 AT5;

W2 BY AT6 AT7 AT8 AT9 AT10;

OUTPUT:

SAMPSTAT STDYX MODINDICES;

INPUT READING TERMINATED NORMALLY

SUMMARY OF ANALYSIS

Number of groups 1

Number of observations 287

Number of dependent variables 10

Number of independent variables 0

Number of continuous latent variables 2

Observed dependent variables

Continuous

AT1 AT2 AT3 AT4 AT5 AT6

AT7 AT8 AT9 AT10

Continuous latent variables

W1 W2

Estimator MLM

Information matrix EXPECTED

Maximum number of iterations 1000

Convergence criterion 0.500D-04

Maximum number of steepest descent iterations 20

Input data file(s)

C:\Users\DELL\Desktop\shu\+AT4+CAR3\3添加CAR3-1.dat

Input data format FREE

SAMPLE STATISTICS

SAMPLE STATISTICS

Means

AT1 AT2 AT3 AT4 AT5

________ ________ ________ ________ ________

4.369 4.317 4.282 4.024 4.401

Means

AT6 AT7 AT8 AT9 AT10

________ ________ ________ ________ ________

4.202 4.136 3.627 4.132 3.969

Covariances

AT1 AT2 AT3 AT4 AT5

________ ________ ________ ________ ________

AT1 0.428

AT2 0.311 0.495

AT3 0.265 0.304 0.607

AT4 0.176 0.208 0.241 1.362

AT5 0.207 0.207 0.218 0.203 0.386

AT6 0.267 0.270 0.319 0.183 0.254

AT7 0.253 0.264 0.282 0.199 0.235

AT8 0.166 0.230 0.255 0.800 0.177

AT9 0.219 0.258 0.283 0.220 0.208

AT10 0.217 0.250 0.309 0.168 0.173

Covariances

AT6 AT7 AT8 AT9 AT10

________ ________ ________ ________ ________

AT6 0.572

AT7 0.450 0.619

AT8 0.236 0.322 1.453

AT9 0.311 0.334 0.192 0.644

AT10 0.299 0.300 0.204 0.391 0.658

Correlations

AT1 AT2 AT3 AT4 AT5

________ ________ ________ ________ ________

AT1 1.000

AT2 0.676 1.000

AT3 0.520 0.555 1.000

AT4 0.230 0.254 0.265 1.000

AT5 0.510 0.474 0.450 0.280 1.000

AT6 0.539 0.508 0.542 0.208 0.539

AT7 0.491 0.476 0.460 0.216 0.480

AT8 0.210 0.271 0.272 0.569 0.237

AT9 0.418 0.456 0.453 0.235 0.417

AT10 0.409 0.439 0.488 0.178 0.343

Correlations

AT6 AT7 AT8 AT9 AT10

________ ________ ________ ________ ________

AT6 1.000

AT7 0.756 1.000

AT8 0.258 0.340 1.000

AT9 0.512 0.529 0.199 1.000

AT10 0.487 0.471 0.209 0.600 1.000

UNIVARIATE SAMPLE STATISTICS

UNIVARIATE HIGHER-ORDER MOMENT DESCRIPTIVE STATISTICS

Variable/ Mean/ Skewness/ Minimum/ % with Percentiles

Sample Size Variance Kurtosis Maximum Min/Max 20%/60% 40%/80% Median

AT1 4.369 -1.152 1.000 0.35% 4.000 4.000 4.000

287.000 0.428 2.950 5.000 44.25% 5.000 5.000

AT2 4.317 -1.073 1.000 0.35% 4.000 4.000 4.000

287.000 0.495 1.979 5.000 42.86% 5.000 5.000

AT3 4.282 -1.068 1.000 0.35% 4.000 4.000 4.000

287.000 0.607 1.198 5.000 44.60% 5.000 5.000

AT4 4.024 -1.152 1.000 4.88% 3.000 4.000 4.000

287.000 1.362 0.354 5.000 44.95% 5.000 5.000

AT5 4.401 -0.621 2.000 0.35% 4.000 4.000 4.000

287.000 0.386 -0.123 5.000 47.04% 5.000 5.000

AT6 4.202 -0.788 1.000 0.35% 4.000 4.000 4.000

287.000 0.572 0.684 5.000 37.98% 4.000 5.000

AT7 4.136 -0.588 2.000 2.79% 4.000 4.000 4.000

287.000 0.619 -0.238 5.000 35.89% 4.000 5.000

AT8 3.627 -0.616 1.000 6.27% 2.000 4.000 4.000

287.000 1.453 -0.621 5.000 27.53% 4.000 5.000

AT9 4.132 -1.011 1.000 1.05% 4.000 4.000 4.000

287.000 0.644 1.613 5.000 34.15% 4.000 5.000

AT10 3.969 -0.492 1.000 0.70% 3.000 4.000 4.000

287.000 0.658 0.179 5.000 27.18% 4.000 5.000

THE MODEL ESTIMATION TERMINATED NORMALLY

MODEL FIT INFORMATION

Number of Free Parameters 31

Loglikelihood

H0 Value -2913.769

H1 Value -2814.926

Information Criteria

Akaike (AIC) 5889.538

Bayesian (BIC) 6002.982

Sample-Size Adjusted BIC 5904.678

(n* = (n + 2) / 24)

**Chi-Square Test of Model Fit**

**Value 149.249***

**Degrees of Freedom 34**

P-Value 0.0000

Scaling Correction Factor 1.3245

for MLM

* The chi-square value for MLM, MLMV, MLR, ULSMV, WLSM and WLSMV cannot be used

for chi-square difference testing in the regular way. MLM, MLR and WLSM

chi-square difference testing is described on the Mplus website. MLMV, WLSMV,

and ULSMV difference testing is done using the DIFFTEST option.

**RMSEA (Root Mean Square Error Of Approximation)**

**Estimate 0.109**

90 Percent C.I. 0.091 0.127

Probability RMSEA <= .05 0.000

**CFI/TLI**

**CFI 0.882**

**TLI 0.844**

Chi-Square Test of Model Fit for the Baseline Model

Value 1025.641

Degrees of Freedom 45

P-Value 0.0000

**SRMR (Standardized Root Mean Square Residual)**

**Value 0.071**

MODEL RESULTS

Two-Tailed

Estimate S.E. Est./S.E. P-Value

W1 BY

AT1 1.000 0.000 999.000 999.000

AT2 1.078 0.078 13.808 0.000

AT3 1.073 0.113 9.495 0.000

AT4 0.783 0.146 5.354 0.000

AT5 0.799 0.099 8.060 0.000

W2 BY

AT6 1.000 0.000 999.000 999.000

AT7 1.017 0.056 18.080 0.000

AT8 0.675 0.108 6.246 0.000

AT9 0.829 0.075 11.059 0.000

AT10 0.791 0.074 10.695 0.000

W2 WITH

W1 0.273 0.034 7.990 0.000

Intercepts

AT1 4.369 0.039 113.138 0.000

AT2 4.317 0.042 103.921 0.000

AT3 4.282 0.046 93.133 0.000

AT4 4.024 0.069 58.424 0.000

AT5 4.401 0.037 119.922 0.000

AT6 4.202 0.045 94.093 0.000

AT7 4.136 0.046 89.044 0.000

AT8 3.627 0.071 50.971 0.000

AT9 4.132 0.047 87.204 0.000

AT10 3.969 0.048 82.912 0.000

Variances

W1 0.263 0.042 6.260 0.000

W2 0.415 0.047 8.883 0.000

Residual Variances

AT1 0.165 0.060 2.762 0.006

AT2 0.190 0.067 2.825 0.005

AT3 0.304 0.047 6.435 0.000

AT4 1.201 0.104 11.498 0.000

AT5 0.219 0.029 7.496 0.000

AT6 0.158 0.032 4.990 0.000

AT7 0.190 0.038 5.021 0.000

AT8 1.265 0.097 13.015 0.000

AT9 0.359 0.063 5.670 0.000

AT10 0.398 0.038 10.526 0.000

STANDARDIZED MODEL RESULTS

STDYX Standardization

Two-Tailed

Estimate S.E. Est./S.E. P-Value

W1 BY

AT1 0.784 0.069 11.364 0.000

AT2 0.786 0.069 11.455 0.000

AT3 0.707 0.043 16.602 0.000

AT4 0.344 0.061 5.674 0.000

AT5 0.659 0.051 12.956 0.000

W2 BY

AT6 0.851 0.028 30.490 0.000

AT7 0.833 0.033 25.351 0.000

AT8 0.360 0.056 6.378 0.000

AT9 0.665 0.054 12.369 0.000

AT10 0.628 0.041 15.341 0.000

W2 WITH

W1 0.827 0.029 28.294 0.000

Intercepts

AT1 6.678 0.466 14.333 0.000

AT2 6.134 0.388 15.799 0.000

AT3 5.497 0.310 17.744 0.000

AT4 3.449 0.154 22.327 0.000

AT5 7.079 0.319 22.214 0.000

AT6 5.554 0.295 18.828 0.000

AT7 5.256 0.233 22.555 0.000

AT8 3.009 0.114 26.435 0.000

AT9 5.147 0.277 18.602 0.000

AT10 4.894 0.182 26.911 0.000

Variances

W1 1.000 0.000 999.000 999.000

W2 1.000 0.000 999.000 999.000

Residual Variances

AT1 0.385 0.108 3.563 0.000

AT2 0.383 0.108 3.552 0.000

AT3 0.501 0.060 8.318 0.000

AT4 0.882 0.042 21.144 0.000

AT5 0.566 0.067 8.441 0.000

AT6 0.275 0.048 5.795 0.000

AT7 0.307 0.055 5.614 0.000

AT8 0.870 0.041 21.372 0.000

AT9 0.558 0.072 7.799 0.000

AT10 0.606 0.051 11.782 0.000

R-SQUARE

Observed Two-Tailed

Variable Estimate S.E. Est./S.E. P-Value

AT1 0.615 0.108 5.682 0.000

AT2 0.617 0.108 5.727 0.000

AT3 0.499 0.060 8.301 0.000

AT4 0.118 0.042 2.837 0.005

AT5 0.434 0.067 6.478 0.000

AT6 0.725 0.048 15.245 0.000

AT7 0.693 0.055 12.676 0.000

AT8 0.130 0.041 3.189 0.001

AT9 0.442 0.072 6.184 0.000

AT10 0.394 0.051 7.670 0.000

QUALITY OF NUMERICAL RESULTS

Condition Number for the Information Matrix 0.264E-02

(ratio of smallest to largest eigenvalue)

MODEL MODIFICATION INDICES

NOTE: Modification indices for direct effects of observed dependent variables

regressed on covariates may not be included. To include these, request

MODINDICES (ALL).

Minimum M.I. value for printing the modification index 10.000

M.I. E.P.C. Std E.P.C. StdYX E.P.C.

WITH Statements

AT2 WITH AT1 14.282 0.074 0.074 0.418

AT7 WITH AT6 27.182 0.134 0.134 0.775

AT8 WITH AT4 58.965 0.654 0.654 0.531

AT10 WITH AT9 27.167 0.151 0.151 0.398

DIAGRAM INFORMATION

Use View Diagram under the Diagram menu in the Mplus Editor to view the diagram.

If running Mplus from the Mplus Diagrammer, the diagram opens automatically.

Diagram output

c:\users\dell\desktop\数据\原始数据\mptext5.dgm

Beginning Time: 14:58:05

Ending Time: 14:58:05

Elapsed Time: 00:00:00

MUTHEN & MUTHEN

3463 Stoner Ave.

Los Angeles, CA 90066

Tel: (310) 391-9971

Fax: (310) 391-8971

Web: www.StatModel.com

Support: Support@StatModel.com

Copyright (c) 1998-2019 Muthen & Muthen

*Coach-Athlete Relationship Questionnaire (CARQ).*

**The Cronbach's a of the CAR Scale is 0.93, among which the Cronbach'sαof closeness, commitment and complementarity are 0.84, 0.82 and 0.84 respectively.**

**
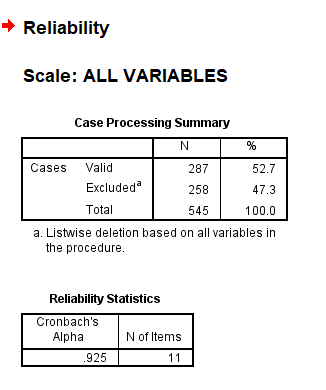
**

**
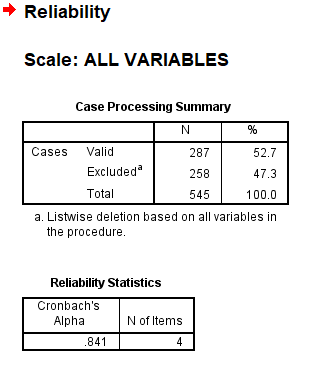
**

**
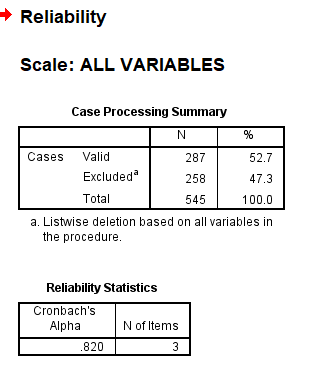
**

**
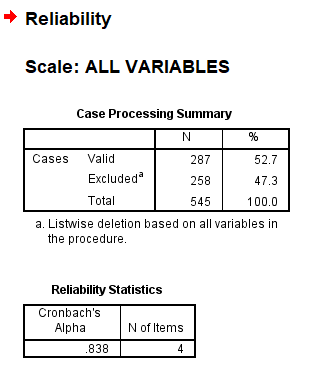
**

**The questionnaire has a total of 11 items, including three dimensions of closeness, commitment and complementarity. CAR scale has a good fit ( χ^2^/df = 2.89, RMSEA = 0.08, SRMR = 0.05,CFI = 0.91, TLI = 0.89 ).**

**
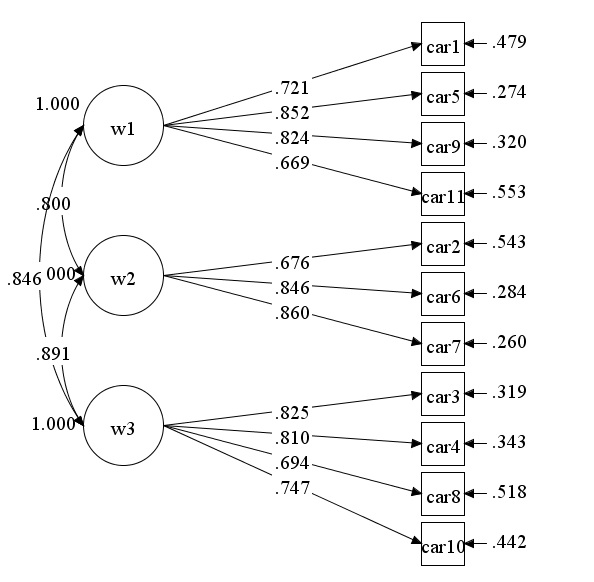
**

**Mplus VERSION 8.3**

**MUTHEN & MUTHEN**

**04/11/2023 5:20 PM**

**INPUT INSTRUCTIONS**

**DATA:**

**FILE IS C:\Users\DELL\Desktop\shu\+AT4+CAR3\3添加CAR3-1.dat;**

**VARIABLE:**

**NAMES ARE xh gender ag syd ds grade ydxm ydnx cdnx AT1 AT2 AT3**

**AT4 AT5 ATw1 ATw1A AT6 AT7 AT8 AT9 AT10 ATw2 ATw2A ATsum**

**ATsumA CAR1 CAR5 CAR9 CAR11 CARw1 CARw1A CAR2 CAR6 CAR7**

**CARw2 CARw2A CAR3 CAR4 CAR8 CAR10 CARw3 CARw3A CARsum**

**CARsumA AE1 AE2 AE3 AE4 AEw1 AEw1A AE5 AE6 AE7 AE8 AEw2**

**AEw2A AE9 AE10 AE11 AE12 AEw3 AEw3A AE13 AE14 AE15 AE16**

**AEw4 AEw4A AEsum AEsumA;**

**USEVARIABLES ARE**

**CAR1 CAR5 CAR9 CAR11 CAR2 CAR6 CAR7**

**CAR3 CAR4 CAR8 CAR10;**

**ANALYSIS:**

**TYPE IS GENERAL;**

**ESTIMATOR IS MLM;**

**MODEL:**

**W1 BY CAR1 CAR5 CAR9 CAR11;**

**W2 BY CAR2 CAR6 CAR7;**

**W3 BY CAR3 CAR4 CAR8 CAR10;**

**OUTPUT:**

**SAMPSTAT STDYX MODINDICES;**

**INPUT READING TERMINATED NORMALLY**

**SUMMARY OF ANALYSIS**

**Number of groups 1**

**Number of observations 287**

**Number of dependent variables 11**

**Number of independent variables 0**

**Number of continuous latent variables 3**

**Observed dependent variables**

**Continuous**

**CAR1 CAR5 CAR9 CAR11 CAR2 CAR6**

**CAR7 CAR3 CAR4 CAR8 CAR10**

**Continuous latent variables**

**W1 W2 W3**

**Estimator MLM**

**Information matrix EXPECTED**

**Maximum number of iterations 1000**

**Convergence criterion 0.500D-04**

**Maximum number of steepest descent iterations 20**

**Input data file(s)**

**C:\Users\DELL\Desktop\shu\+AT4+CAR3\3添加CAR3-1.dat**

**Input data format FREE**

**SAMPLE STATISTICS**

**SAMPLE STATISTICS**

**Means**

**CAR1 CAR5 CAR9 CAR11 CAR2**

**________ ________ ________ ________ ________**

**4.599 4.638 4.662 4.592 4.369**

**Means**

**CAR6 CAR7 CAR3 CAR4 CAR8**

**________ ________ ________ ________ ________**

**4.592 4.526 4.540 4.446 4.369**

**Means**

**CAR10**

**________**

**4.648**

**Covariances**

**CAR1 CAR5 CAR9 CAR11 CAR2**

**________ ________ ________ ________ ________**

**CAR1 0.414**

**CAR5 0.210 0.287**

**CAR9 0.213 0.216 0.307**

**CAR11 0.192 0.173 0.207 0.409**

**CAR2 0.239 0.179 0.160 0.158 0.498**

**CAR6 0.182 0.197 0.165 0.179 0.224**

**CAR7 0.183 0.215 0.188 0.190 0.248**

**CAR3 0.237 0.199 0.186 0.196 0.264**

**CAR4 0.189 0.179 0.161 0.206 0.267**

**CAR8 0.190 0.214 0.184 0.196 0.250**

**CAR10 0.176 0.169 0.188 0.233 0.182**

**Covariances**

**CAR6 CAR7 CAR3 CAR4 CAR8**

**________ ________ ________ ________ ________**

**CAR6 0.360**

**CAR7 0.288 0.403**

**CAR3 0.217 0.211 0.374**

**CAR4 0.217 0.225 0.299 0.407**

**CAR8 0.286 0.335 0.243 0.271 0.616**

**CAR10 0.188 0.217 0.194 0.213 0.231**

**Covariances**

**CAR10**

**________**

**CAR10 0.319**

**Correlations**

**CAR1 CAR5 CAR9 CAR11 CAR2**

**________ ________ ________ ________ ________**

**CAR1 1.000**

**CAR5 0.610 1.000**

**CAR9 0.597 0.726 1.000**

**CAR11 0.467 0.505 0.584 1.000**

**CAR2 0.525 0.474 0.408 0.349 1.000**

**CAR6 0.470 0.614 0.497 0.466 0.529**

**CAR7 0.448 0.633 0.535 0.469 0.554**

**CAR3 0.603 0.608 0.549 0.501 0.612**

**CAR4 0.460 0.524 0.456 0.505 0.594**

**CAR8 0.376 0.509 0.423 0.390 0.452**

**CAR10 0.485 0.558 0.600 0.645 0.458**

**Correlations**

**CAR6 CAR7 CAR3 CAR4 CAR8**

**________ ________ ________ ________ ________**

**CAR6 1.000**

**CAR7 0.756 1.000**

**CAR3 0.591 0.543 1.000**

**CAR4 0.566 0.556 0.767 1.000**

**CAR8 0.608 0.673 0.506 0.541 1.000**

**CAR10 0.554 0.604 0.561 0.590 0.521**

**Correlations**

**CAR10**

**________**

**CAR10 1.000**

**UNIVARIATE SAMPLE STATISTICS**

**UNIVARIATE HIGHER-ORDER MOMENT DESCRIPTIVE STATISTICS**

**Variable/ Mean/ Skewness/ Minimum/ % with Percentiles**

**Sample Size Variance Kurtosis Maximum Min/Max 20%/60% 40%/80% Median**

**CAR1 4.599 -1.902 1.000 0.35% 4.000 5.000 5.000**

**287.000 0.414 4.828 5.000 66.55% 5.000 5.000**

**CAR5 4.638 -1.244 2.000 0.35% 4.000 5.000 5.000**

**287.000 0.287 1.349 5.000 66.20% 5.000 5.000**

**CAR9 4.662 -1.523 2.000 0.35% 4.000 5.000 5.000**

**287.000 0.307 2.020 5.000 70.03% 5.000 5.000**

**CAR11 4.592 -1.868 1.000 0.35% 4.000 5.000 5.000**

**287.000 0.409 4.835 5.000 65.51% 5.000 5.000**

**CAR2 4.369 -1.019 1.000 0.35% 4.000 4.000 4.000**

**287.000 0.498 1.325 5.000 48.43% 5.000 5.000**

**CAR6 4.592 -1.569 1.000 0.35% 4.000 5.000 5.000**

**287.000 0.360 3.819 5.000 64.11% 5.000 5.000**

**CAR7 4.526 -1.244 2.000 1.05% 4.000 4.000 5.000**

**287.000 0.403 1.492 5.000 59.23% 5.000 5.000**

**CAR3 4.540 -1.251 2.000 1.05% 4.000 4.000 5.000**

**287.000 0.374 1.788 5.000 59.23% 5.000 5.000**

**CAR4 4.446 -0.963 2.000 1.05% 4.000 4.000 5.000**

**287.000 0.407 0.934 5.000 51.57% 5.000 5.000**

**CAR8 4.369 -1.184 2.000 3.48% 4.000 4.000 5.000**

**287.000 0.616 0.935 5.000 52.61% 5.000 5.000**

**CAR10 4.648 -1.471 2.000 0.35% 4.000 5.000 5.000**

**287.000 0.319 1.795 5.000 68.99% 5.000 5.000**

**THE MODEL ESTIMATION TERMINATED NORMALLY**

**MODEL FIT INFORMATION**

**Number of Free Parameters 36**

**Loglikelihood**

**H0 Value -2073.190**

**H1 Value -1948.005**

**Information Criteria**

**Akaike (AIC) 4218.379**

**Bayesian (BIC) 4350.121**

**Sample-Size Adjusted BIC 4235.961**

**(n* = (n + 2) / 24)**

**Chi-Square Test of Model Fit**

**Value 113.834***

**Degrees of Freedom 41**

**P-Value 0.0000**

**Scaling Correction Factor 2.1994**

**for MLM**

*** The chi-square value for MLM, MLMV, MLR, ULSMV, WLSM and WLSMV cannot be used**

**for chi-square difference testing in the regular way. MLM, MLR and WLSM**

**chi-square difference testing is described on the Mplus website. MLMV, WLSMV,**

**and ULSMV difference testing is done using the DIFFTEST option.**

**RMSEA (Root Mean Square Error Of Approximation)**

**Estimate 0.079**

**90 Percent C.I. 0.062 0.096**

**Probability RMSEA <= .05 0.004**

**CFI/TLI**

**CFI 0.920**

**TLI 0.892**

**Chi-Square Test of Model Fit for the Baseline Model**

**Value 961.026**

**Degrees of Freedom 55**

**P-Value 0.0000**

**SRMR (Standardized Root Mean Square Residual)**

**Value 0.054**

**MODEL RESULTS**

**Two-Tailed**

**Estimate S.E. Est./S.E. P-Value**

**W1 BY**

**CAR1 1.000 0.000 999.000 999.000**

**CAR5 0.983 0.089 11.066 0.000**

**CAR9 0.984 0.087 11.365 0.000**

**CAR11 0.920 0.093 9.937 0.000**

**W2 BY**

**CAR2 1.000 0.000 999.000 999.000**

**CAR6 1.064 0.125 8.498 0.000**

**CAR7 1.145 0.097 11.808 0.000**

**W3 BY**

**CAR3 1.000 0.000 999.000 999.000**

**CAR4 1.025 0.058 17.804 0.000**

**CAR8 1.080 0.086 12.552 0.000**

**CAR10 0.836 0.080 10.417 0.000**

**W2 WITH**

**W1 0.177 0.026 6.693 0.000**

**W3 WITH**

**W1 0.198 0.026 7.489 0.000**

**W2 0.215 0.027 8.040 0.000**

**Intercepts**

**CAR1 4.599 0.038 121.045 0.000**

**CAR5 4.638 0.032 146.704 0.000**

**CAR9 4.662 0.033 142.457 0.000**

**CAR11 4.592 0.038 121.692 0.000**

**CAR2 4.369 0.042 104.920 0.000**

**CAR6 4.592 0.035 129.676 0.000**

**CAR7 4.526 0.037 120.842 0.000**

**CAR3 4.540 0.036 125.796 0.000**

**CAR4 4.446 0.038 118.010 0.000**

**CAR8 4.369 0.046 94.296 0.000**

**CAR10 4.648 0.033 139.492 0.000**

**Variances**

**W1 0.216 0.037 5.835 0.000**

**W2 0.228 0.034 6.678 0.000**

**W3 0.255 0.037 6.892 0.000**

**Residual Variances**

**CAR1 0.199 0.060 3.321 0.001**

**CAR5 0.078 0.017 4.709 0.000**

**CAR9 0.098 0.019 5.115 0.000**

**CAR11 0.226 0.058 3.865 0.000**

**CAR2 0.270 0.035 7.663 0.000**

**CAR6 0.102 0.032 3.191 0.001**

**CAR7 0.105 0.017 6.154 0.000**

**CAR3 0.119 0.018 6.565 0.000**

**CAR4 0.140 0.016 8.628 0.000**

**CAR8 0.319 0.037 8.671 0.000**

**CAR10 0.141 0.017 8.499 0.000**

**STANDARDIZED MODEL RESULTS**

**STDYX Standardization**

**Two-Tailed**

**Estimate S.E. Est./S.E. P-Value**

**W1 BY**

**CAR1 0.721 0.067 10.825 0.000**

**CAR5 0.852 0.034 25.271 0.000**

**CAR9 0.824 0.030 27.091 0.000**

**CAR11 0.669 0.066 10.194 0.000**

**W2 BY**

**CAR2 0.676 0.037 18.481 0.000**

**CAR6 0.846 0.042 19.950 0.000**

**CAR7 0.860 0.019 44.295 0.000**

**W3 BY**

**CAR3 0.825 0.029 28.459 0.000**

**CAR4 0.810 0.027 29.830 0.000**

**CAR8 0.694 0.037 18.741 0.000**

**CAR10 0.747 0.029 25.856 0.000**

**W2 WITH**

**W1 0.800 0.057 14.031 0.000**

**W3 WITH**

**W1 0.846 0.055 15.487 0.000**

**W2 0.891 0.032 27.848 0.000**

**Intercepts**

**CAR1 7.145 0.569 12.561 0.000**

**CAR5 8.660 0.494 17.538 0.000**

**CAR9 8.409 0.524 16.049 0.000**

**CAR11 7.183 0.559 12.861 0.000**

**CAR2 6.193 0.339 18.248 0.000**

**CAR6 7.655 0.587 13.037 0.000**

**CAR7 7.133 0.410 17.409 0.000**

**CAR3 7.425 0.431 17.231 0.000**

**CAR4 6.966 0.355 19.635 0.000**

**CAR8 5.566 0.310 17.960 0.000**

**CAR10 8.234 0.476 17.307 0.000**

**Variances**

**W1 1.000 0.000 999.000 999.000**

**W2 1.000 0.000 999.000 999.000**

**W3 1.000 0.000 999.000 999.000**

**Residual Variances**

**CAR1 0.479 0.096 4.985 0.000**

**CAR5 0.274 0.057 4.761 0.000**

**CAR9 0.320 0.050 6.386 0.000**

**CAR11 0.553 0.088 6.302 0.000**

**CAR2 0.543 0.049 10.976 0.000**

**CAR6 0.284 0.072 3.964 0.000**

**CAR7 0.260 0.033 7.764 0.000**

**CAR3 0.319 0.048 6.664 0.000**

**CAR4 0.343 0.044 7.802 0.000**

**CAR8 0.518 0.051 10.085 0.000**

**CAR10 0.442 0.043 10.220 0.000**

**R-SQUARE**

**Observed Two-Tailed**

**Variable Estimate S.E. Est./S.E. P-Value**

**CAR1 0.521 0.096 5.413 0.000**

**CAR5 0.726 0.057 12.635 0.000**

**CAR9 0.680 0.050 13.545 0.000**

**CAR11 0.447 0.088 5.097 0.000**

**CAR2 0.457 0.049 9.241 0.000**

**CAR6 0.716 0.072 9.975 0.000**

**CAR7 0.740 0.033 22.148 0.000**

**CAR3 0.681 0.048 14.230 0.000**

**CAR4 0.657 0.044 14.915 0.000**

**CAR8 0.482 0.051 9.371 0.000**

**CAR10 0.558 0.043 12.928 0.000**

**QUALITY OF NUMERICAL RESULTS**

**Condition Number for the Information Matrix 0.119E-02**

**(ratio of smallest to largest eigenvalue)**

**MODEL MODIFICATION INDICES**

**NOTE: Modification indices for direct effects of observed dependent variables**

**regressed on covariates may not be included. To include these, request**

**MODINDICES (ALL).**

**Minimum M.I. value for printing the modification index 10.000**

**M.I. E.P.C. Std E.P.C. StdYX E.P.C.**

**BY Statements**

**W2 BY CAR8 16.822 1.650 0.787 1.003**

**W3 BY CAR2 10.260 1.140 0.575 0.816**

**WITH Statements**

**CAR3 WITH CAR7 11.971 -0.048 -0.048 -0.427**

**CAR4 WITH CAR3 25.966 0.087 0.087 0.672**

**CAR8 WITH CAR7 13.284 0.074 0.074 0.406**

**CAR10 WITH CAR11 14.248 0.066 0.066 0.370**

**DIAGRAM INFORMATION**

**Use View Diagram under the Diagram menu in the Mplus Editor to view the diagram.**

**If running Mplus from the Mplus Diagrammer, the diagram opens automatically.**

**Diagram output**

**c:\users\dell\desktop\数据\原始数据\mptext6.dgm**

**Beginning Time: 17:20:50**

**Ending Time: 17:20:51**

**Elapsed Time: 00:00:01**

**MUTHEN & MUTHEN**

**3463 Stoner Ave.**

**Los Angeles, CA 90066**

**Tel: (310) 391-9971**

**Fax: (310) 391-8971**

**Web: www.StatModel.com**

**Support: Support@StatModel.com**

**Copyright (c) 1998-2019 Muthen & Muthen**
