# Supplementary figures and images for: Association between coach-athlete relationship and athlete engagement in Chinese team sports: The mediating effect of thriving
Source: PLoS One. 2023 Aug 17;18(8):e0289979. doi: 10.1371/journal.pone.0289979 (PMC10434943; doi:10.1371/journal.pone.0289979)

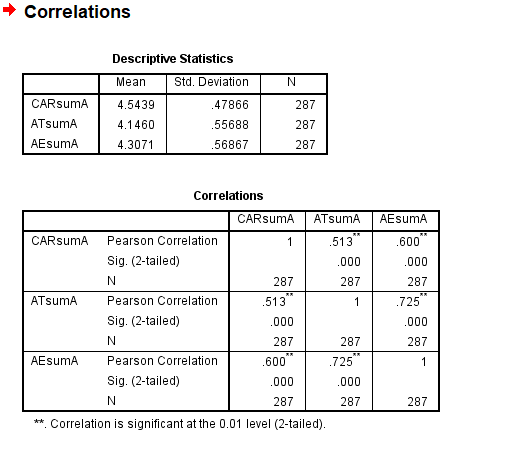

Supplement: S2 File — (DOCX) [file pone.0289979.s002.docx]
