# Supplementary material for: Association between coach-athlete relationship and athlete engagement in Chinese team sports: The mediating effect of thriving
Source: PLoS One. 2023 Aug 17;18(8):e0289979. doi: 10.1371/journal.pone.0289979 (PMC10434943; doi:10.1371/journal.pone.0289979)

**Table 4. CAR constructs predicting relative contribution of athlete engagement.**


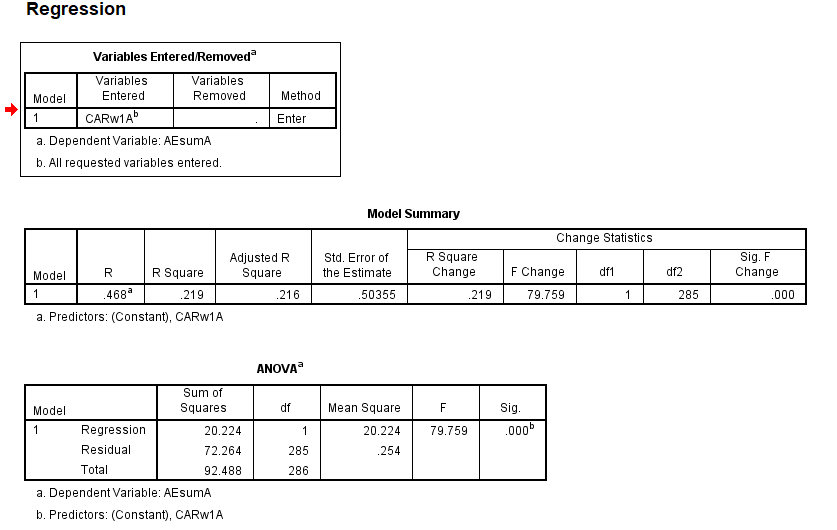


| **Coefficients^a^** | | | | | | |
| --- | --- | --- | --- | --- | --- | --- |
| Model | | Unstandardized Coefficients | | Standardized Coefficients | t | Sig. |
|  |  | B | Std. Error | Beta |  |  |
| 1 | (Constant) | 1.802 | .282 |  | 6.387 | .000 |
|  | CARw1A | .542 | .061 | .468 | 8.931 | .000 |
| a. Dependent Variable: AEsumA | | | | | | |


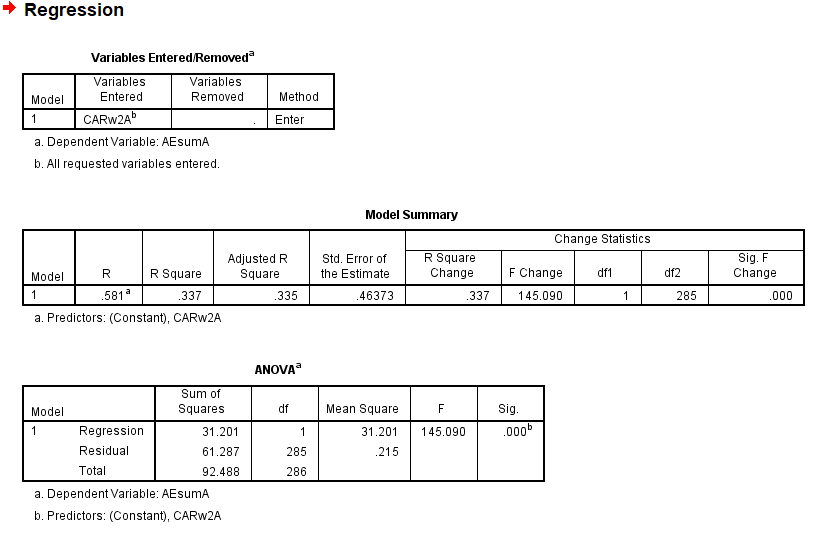


| **Coefficients^a^** | | | | | | |
| --- | --- | --- | --- | --- | --- | --- |
| Model | | Unstandardized Coefficients | | Standardized Coefficients | t | Sig. |
|  |  | B | Std. Error | Beta |  |  |
| 1 | (Constant) | 1.640 | .223 |  | 7.348 | .000 |
|  | CARw2A | .593 | .049 | .581 | 12.045 | .000 |
| a. Dependent Variable: AEsumA | | | | | | |


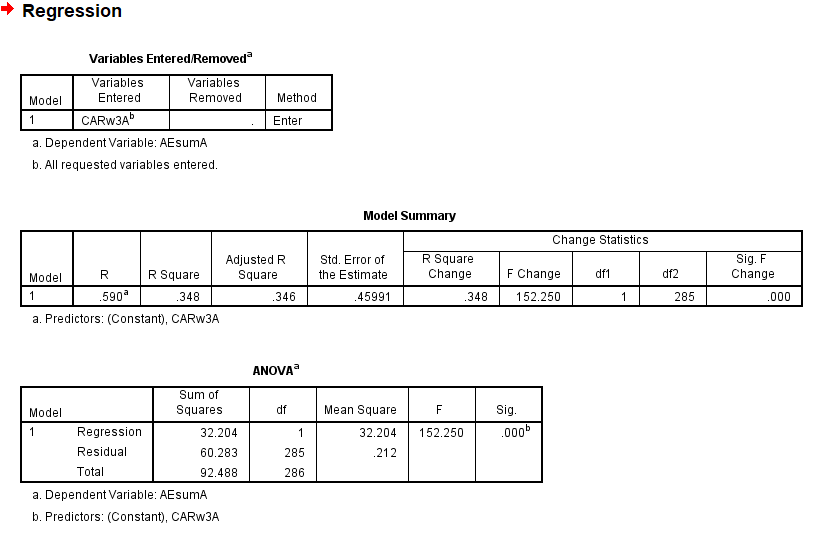


| **Coefficients^a^** | | | | | | |
| --- | --- | --- | --- | --- | --- | --- |
| Model | | Unstandardized Coefficients | | Standardized Coefficients | t | Sig. |
|  |  | B | Std. Error | Beta |  |  |
| 1 | (Constant) | 1.500 | .229 |  | 6.549 | .000 |
|  | CARw3A | .624 | .051 | .590 | 12.339 | .000 |
| a. Dependent Variable: AEsumA | | | | | | |


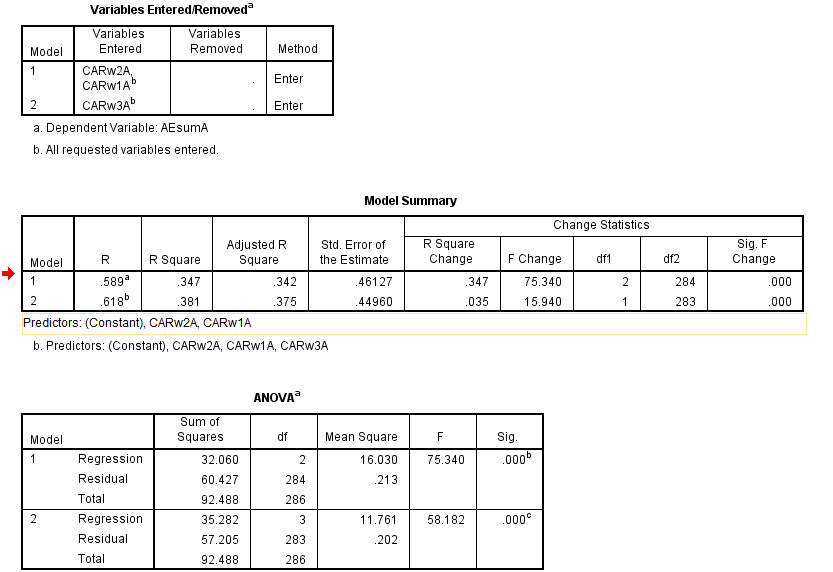


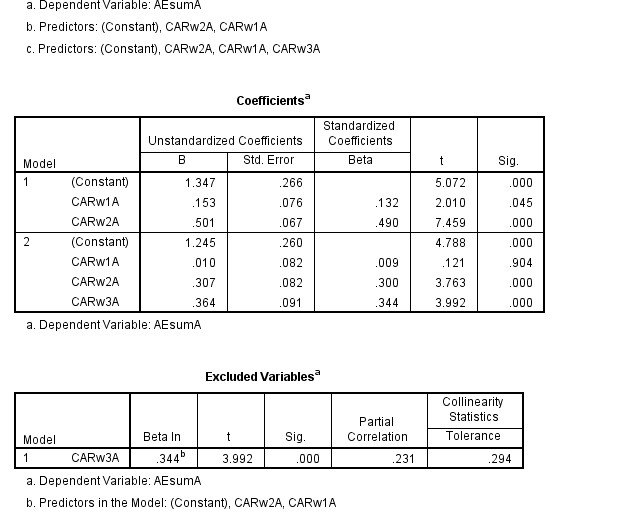


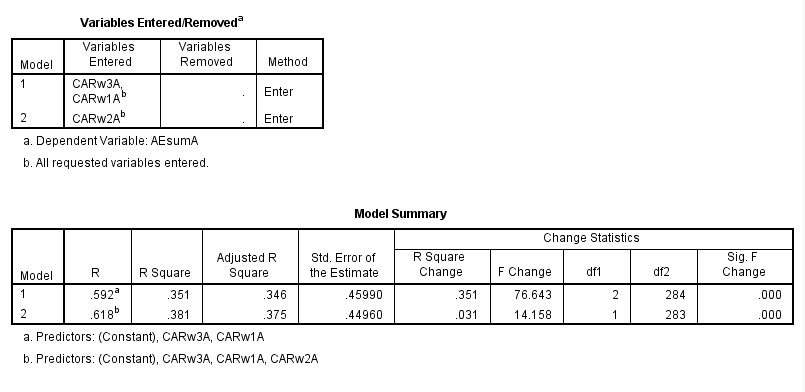


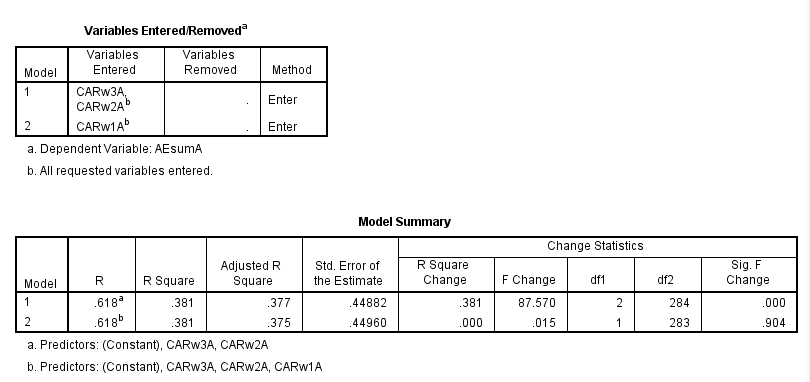


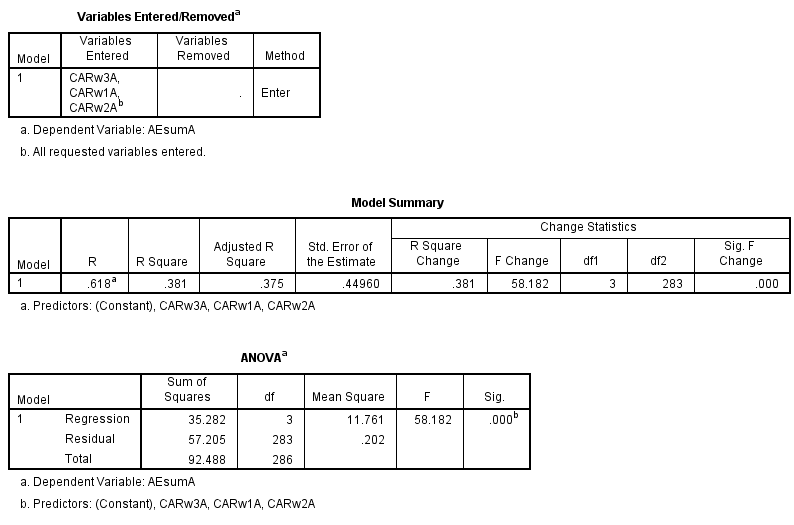

Supplement: S3 File — (DOCX) [file pone.0289979.s003.docx]
