# Supplementary material for: Association between coach-athlete relationship and athlete engagement in Chinese team sports: The mediating effect of thriving
Source: PLoS One. 2023 Aug 17;18(8):e0289979. doi: 10.1371/journal.pone.0289979 (PMC10434943; doi:10.1371/journal.pone.0289979)
Supplement: S4 File — (DOCX) [file pone.0289979.s004.docx]

STANDARDIZED MODEL RESULTS

STDYX Standardization

Two-Tailed

Estimate S.E. Est./S.E. P-Value

CAR BY

CARW1A 0.792 0.050 15.807 0.000

CARW2A 0.868 0.027 32.690 0.000

CARW3A 0.922 0.022 42.584 0.000

TH BY

ATW1A 0.839 0.026 31.876 0.000

ATW2A 0.861 0.027 31.548 0.000

AE BY

AEW1A 0.792 0.031 25.161 0.000

AEW2A 0.853 0.022 38.042 0.000

AEW3A 0.911 0.014 65.305 0.000

AEW4A 0.872 0.019 45.844 0.000

AE ON

CAR 0.288 0.068 4.264 0.000

TH 0.654 0.065 10.139 0.000

TH ON

CAR 0.581 0.055 10.597 0.000

Intercepts

CARW1A 9.438 0.501 18.846 0.000

CARW2A 8.090 0.369 21.920 0.000

CARW3A 8.379 0.421 19.911 0.000

ATW1A 7.583 0.316 23.979 0.000

ATW2A 6.335 0.248 25.516 0.000

AEW1A 6.517 0.266 24.489 0.000

AEW2A 6.211 0.324 19.157 0.000

AEW3A 7.843 0.315 24.917 0.000

AEW4A 6.717 0.356 18.885 0.000

Variances

CAR 1.000 0.000 999.000 999.000

Residual Variances

CARW1A 0.373 0.078 4.766 0.000

CARW2A 0.246 0.046 5.339 0.000

CARW3A 0.149 0.040 3.752 0.000

ATW1A 0.296 0.044 6.704 0.000

ATW2A 0.259 0.047 5.544 0.000

AEW1A 0.373 0.050 7.535 0.000

AEW2A 0.272 0.038 7.110 0.000

AEW3A 0.170 0.025 6.703 0.000

AEW4A 0.240 0.033 7.259 0.000

TH 0.662 0.063 10.437 0.000

AE 0.270 0.046 5.808 0.000

STDY Standardization

Two-Tailed

Estimate S.E. Est./S.E. P-Value

CAR BY

CARW1A 0.792 0.050 15.807 0.000

CARW2A 0.868 0.027 32.690 0.000

CARW3A 0.922 0.022 42.584 0.000

TH BY

ATW1A 0.839 0.026 31.876 0.000

ATW2A 0.861 0.027 31.548 0.000

AE BY

AEW1A 0.792 0.031 25.161 0.000

AEW2A 0.853 0.022 38.042 0.000

AEW3A 0.911 0.014 65.305 0.000

AEW4A 0.872 0.019 45.844 0.000

AE ON

CAR 0.288 0.068 4.264 0.000

TH 0.654 0.065 10.139 0.000

TH ON

CAR 0.581 0.055 10.597 0.000

Intercepts

CARW1A 9.438 0.501 18.846 0.000

CARW2A 8.090 0.369 21.920 0.000

CARW3A 8.379 0.421 19.911 0.000

ATW1A 7.583 0.316 23.979 0.000

ATW2A 6.335 0.248 25.516 0.000

AEW1A 6.517 0.266 24.489 0.000

AEW2A 6.211 0.324 19.157 0.000

AEW3A 7.843 0.315 24.917 0.000

AEW4A 6.717 0.356 18.885 0.000

Variances

CAR 1.000 0.000 999.000 999.000

Residual Variances

CARW1A 0.373 0.078 4.766 0.000

CARW2A 0.246 0.046 5.339 0.000

CARW3A 0.149 0.040 3.752 0.000

ATW1A 0.296 0.044 6.704 0.000

ATW2A 0.259 0.047 5.544 0.000

AEW1A 0.373 0.050 7.535 0.000

AEW2A 0.272 0.038 7.110 0.000

AEW3A 0.170 0.025 6.703 0.000

AEW4A 0.240 0.033 7.259 0.000

TH 0.662 0.063 10.437 0.000

AE 0.270 0.046 5.808 0.000

STD Standardization

Two-Tailed

Estimate S.E. Est./S.E. P-Value

CAR BY

CARW1A 0.388 0.024 16.501 0.000

CARW2A 0.483 0.023 20.687 0.000

CARW3A 0.495 0.025 19.802 0.000

TH BY

ATW1A 0.474 0.027 17.710 0.000

ATW2A 0.545 0.031 17.678 0.000

AE BY

AEW1A 0.517 0.032 16.314 0.000

AEW2A 0.579 0.030 19.141 0.000

AEW3A 0.510 0.022 22.932 0.000

AEW4A 0.567 0.029 19.632 0.000

AE ON

CAR 0.288 0.068 4.264 0.000

TH 0.654 0.065 10.139 0.000

TH ON

CAR 0.581 0.055 10.597 0.000

Intercepts

CARW1A 4.623 0.029 158.738 0.000

CARW2A 4.496 0.033 136.208 0.000

CARW3A 4.501 0.032 141.915 0.000

ATW1A 4.279 0.033 127.999 0.000

ATW2A 4.014 0.038 105.671 0.000

AEW1A 4.253 0.039 108.755 0.000

AEW2A 4.213 0.040 105.704 0.000

AEW3A 4.390 0.033 132.472 0.000

AEW4A 4.370 0.038 114.408 0.000

Variances

CAR 1.000 0.000 999.000 999.000

Residual Variances

CARW1A 0.089 0.024 3.668 0.000

CARW2A 0.076 0.016 4.761 0.000

CARW3A 0.043 0.012 3.535 0.000

ATW1A 0.094 0.014 6.909 0.000

ATW2A 0.104 0.017 6.036 0.000

AEW1A 0.159 0.021 7.732 0.000

AEW2A 0.125 0.022 5.634 0.000

AEW3A 0.053 0.008 6.945 0.000

AEW4A 0.102 0.017 5.911 0.000

TH 0.662 0.063 10.437 0.000

AE 0.270 0.046 5.808 0.000

R-SQUARE

Observed Two-Tailed

Variable Estimate S.E. Est./S.E. P-Value

CARW1A 0.627 0.078 8.018 0.000

CARW2A 0.754 0.046 16.368 0.000

CARW3A 0.851 0.040 21.350 0.000

ATW1A 0.704 0.044 15.976 0.000

ATW2A 0.741 0.047 15.844 0.000

AEW1A 0.627 0.050 12.647 0.000

AEW2A 0.728 0.038 19.064 0.000

AEW3A 0.830 0.025 32.716 0.000

AEW4A 0.760 0.033 22.963 0.000

Latent Two-Tailed

Variable Estimate S.E. Est./S.E. P-Value

TH 0.338 0.063 5.330 0.000

AE 0.730 0.046 15.738 0.000

TOTAL, TOTAL INDIRECT, SPECIFIC INDIRECT, AND DIRECT EFFECTS

Two-Tailed

Estimate S.E. Est./S.E. P-Value

Effects from CAR to AE

Total 0.891 0.102 8.722 0.000

Total indirect 0.507 0.070 7.219 0.000

Specific indirect 1

AE

TH

CAR 0.507 0.070 7.219 0.000

Direct

AE

CAR 0.384 0.098 3.937 0.000

STANDARDIZED TOTAL, TOTAL INDIRECT, SPECIFIC INDIRECT, AND DIRECT EFFECTS

STDYX Standardization

Two-Tailed

Estimate S.E. Est./S.E. P-Value

Effects from CAR to AE

Total 0.669 0.053 12.596 0.000

Total indirect 0.380 0.045 8.474 0.000

Specific indirect 1

AE

TH

CAR 0.380 0.045 8.474 0.000

Direct

AE

CAR 0.288 0.068 4.264 0.000

STDY Standardization

Two-Tailed

Estimate S.E. Est./S.E. P-Value

Effects from CAR to AE

Total 0.669 0.053 12.596 0.000

Total indirect 0.380 0.045 8.474 0.000

Specific indirect 1

AE

TH

CAR 0.380 0.045 8.474 0.000

Direct

AE

CAR 0.288 0.068 4.264 0.000

STD Standardization

Two-Tailed

Estimate S.E. Est./S.E. P-Value

Effects from CAR to AE

Total 0.669 0.053 12.596 0.000

Total indirect 0.380 0.045 8.474 0.000

Specific indirect 1

AE

TH

CAR 0.380 0.045 8.474 0.000

Direct

AE

CAR 0.288 0.068 4.264 0.000

CONFIDENCE INTERVALS OF MODEL RESULTS

Lower .5% Lower 2.5% Lower 5% Estimate Upper 5% Upper 2.5% Upper .5%

CAR BY

CARW1A 1.000 1.000 1.000 1.000 1.000 1.000 1.000

CARW2A 1.055 1.095 1.120 1.244 1.397 1.427 1.500

CARW3A 1.101 1.141 1.160 1.277 1.423 1.457 1.520

TH BY

ATW1A 1.000 1.000 1.000 1.000 1.000 1.000 1.000

ATW2A 0.985 1.021 1.041 1.152 1.282 1.307 1.368

AE BY

AEW1A 1.000 1.000 1.000 1.000 1.000 1.000 1.000

AEW2A 0.940 0.980 1.000 1.120 1.264 1.297 1.365

AEW3A 0.845 0.876 0.893 0.987 1.099 1.122 1.178

AEW4A 0.931 0.974 0.991 1.098 1.211 1.235 1.295

AE ON

CAR 0.157 0.209 0.235 0.384 0.554 0.591 0.672

TH 0.493 0.551 0.579 0.714 0.852 0.880 0.937

TH ON

CAR 0.517 0.563 0.587 0.710 0.842 0.871 0.932

Intercepts

CARW1A 4.548 4.564 4.574 4.623 4.670 4.679 4.695

CARW2A 4.409 4.430 4.441 4.496 4.548 4.561 4.582

CARW3A 4.421 4.440 4.449 4.501 4.554 4.565 4.584

ATW1A 4.187 4.211 4.223 4.279 4.332 4.342 4.364

ATW2A 3.918 3.942 3.953 4.014 4.077 4.090 4.116

AEW1A 4.153 4.177 4.189 4.253 4.320 4.332 4.355

AEW2A 4.109 4.136 4.147 4.213 4.279 4.291 4.315

AEW3A 4.305 4.325 4.337 4.390 4.445 4.456 4.477

AEW4A 4.273 4.297 4.308 4.370 4.434 4.446 4.469

Variances

CAR 0.107 0.116 0.121 0.150 0.181 0.188 0.202

Residual Variances

CARW1A 0.047 0.055 0.059 0.089 0.144 0.153 0.175

CARW2A 0.041 0.048 0.052 0.076 0.105 0.111 0.123

CARW3A 0.015 0.021 0.025 0.043 0.065 0.070 0.079

ATW1A 0.062 0.069 0.074 0.094 0.118 0.123 0.133

ATW2A 0.061 0.072 0.077 0.104 0.134 0.140 0.150

AEW1A 0.110 0.121 0.127 0.159 0.195 0.203 0.214

AEW2A 0.079 0.088 0.093 0.125 0.168 0.176 0.193

AEW3A 0.036 0.039 0.041 0.053 0.067 0.070 0.075

AEW4A 0.064 0.072 0.077 0.102 0.134 0.141 0.152

TH 0.100 0.111 0.116 0.148 0.187 0.196 0.210

AE 0.043 0.049 0.053 0.072 0.098 0.102 0.113

CONFIDENCE INTERVALS OF STANDARDIZED MODEL RESULTS

STDYX Standardization

Lower .5% Lower 2.5% Lower 5% Estimate Upper 5% Upper 2.5% Upper .5%

CAR BY

CARW1A 0.632 0.668 0.689 0.792 0.859 0.868 0.886

CARW2A 0.793 0.810 0.822 0.868 0.908 0.915 0.928

CARW3A 0.860 0.876 0.885 0.922 0.956 0.962 0.973

TH BY

ATW1A 0.760 0.782 0.791 0.839 0.878 0.886 0.901

ATW2A 0.782 0.803 0.813 0.861 0.902 0.909 0.925

AE BY

AEW1A 0.699 0.725 0.737 0.792 0.840 0.849 0.864

AEW2A 0.789 0.806 0.814 0.853 0.888 0.893 0.906

AEW3A 0.869 0.881 0.886 0.911 0.932 0.936 0.943

AEW4A 0.816 0.829 0.836 0.872 0.899 0.904 0.915

AE ON

CAR 0.122 0.162 0.182 0.288 0.405 0.427 0.473

TH 0.468 0.517 0.542 0.654 0.753 0.772 0.806

TH ON

CAR 0.430 0.468 0.486 0.581 0.666 0.682 0.712

Intercepts

CARW1A 8.210 8.492 8.635 9.438 10.282 10.461 10.774

CARW2A 7.237 7.412 7.513 8.090 8.726 8.872 9.130

CARW3A 7.397 7.621 7.727 8.379 9.088 9.246 9.553

ATW1A 6.815 6.977 7.074 7.583 8.115 8.224 8.409

ATW2A 5.729 5.869 5.938 6.335 6.750 6.843 6.997

AEW1A 5.898 6.024 6.091 6.517 6.976 7.063 7.236

AEW2A 5.421 5.602 5.681 6.211 6.740 6.844 7.037

AEW3A 7.054 7.203 7.291 7.843 8.327 8.441 8.611

AEW4A 5.901 6.074 6.163 6.717 7.330 7.459 7.739

Variances

CAR 1.000 1.000 1.000 1.000 1.000 1.000 1.000

Residual Variances

CARW1A 0.215 0.247 0.263 0.373 0.526 0.553 0.600

CARW2A 0.139 0.162 0.176 0.246 0.325 0.344 0.370

CARW3A 0.053 0.075 0.086 0.149 0.217 0.232 0.261

ATW1A 0.189 0.215 0.229 0.296 0.374 0.389 0.423

ATW2A 0.144 0.173 0.186 0.259 0.340 0.356 0.387

AEW1A 0.253 0.279 0.294 0.373 0.456 0.474 0.508

AEW2A 0.179 0.202 0.211 0.272 0.338 0.350 0.376

AEW3A 0.110 0.125 0.131 0.170 0.215 0.224 0.243

AEW4A 0.163 0.182 0.191 0.240 0.300 0.313 0.334

TH 0.493 0.535 0.556 0.662 0.764 0.781 0.813

AE 0.163 0.188 0.201 0.270 0.353 0.370 0.404

STDY Standardization

Lower .5% Lower 2.5% Lower 5% Estimate Upper 5% Upper 2.5% Upper .5%

CAR BY

CARW1A 0.632 0.668 0.689 0.792 0.859 0.868 0.886

CARW2A 0.793 0.810 0.822 0.868 0.908 0.915 0.928

CARW3A 0.860 0.876 0.885 0.922 0.956 0.962 0.973

TH BY

ATW1A 0.760 0.782 0.791 0.839 0.878 0.886 0.901

ATW2A 0.782 0.803 0.813 0.861 0.902 0.909 0.925

AE BY

AEW1A 0.699 0.725 0.737 0.792 0.840 0.849 0.864

AEW2A 0.789 0.806 0.814 0.853 0.888 0.893 0.906

AEW3A 0.869 0.881 0.886 0.911 0.932 0.936 0.943

AEW4A 0.816 0.829 0.836 0.872 0.899 0.904 0.915

AE ON

CAR 0.122 0.162 0.182 0.288 0.405 0.427 0.473

TH 0.468 0.517 0.542 0.654 0.753 0.772 0.806

TH ON

CAR 0.430 0.468 0.486 0.581 0.666 0.682 0.712

Intercepts

CARW1A 8.210 8.492 8.635 9.438 10.282 10.461 10.774

CARW2A 7.237 7.412 7.513 8.090 8.726 8.872 9.130

CARW3A 7.397 7.621 7.727 8.379 9.088 9.246 9.553

ATW1A 6.815 6.977 7.074 7.583 8.115 8.224 8.409

ATW2A 5.729 5.869 5.938 6.335 6.750 6.843 6.997

AEW1A 5.898 6.024 6.091 6.517 6.976 7.063 7.236

AEW2A 5.421 5.602 5.681 6.211 6.740 6.844 7.037

AEW3A 7.054 7.203 7.291 7.843 8.327 8.441 8.611

AEW4A 5.901 6.074 6.163 6.717 7.330 7.459 7.739

Variances

CAR 1.000 1.000 1.000 1.000 1.000 1.000 1.000

Residual Variances

CARW1A 0.215 0.247 0.263 0.373 0.526 0.553 0.600

CARW2A 0.139 0.162 0.176 0.246 0.325 0.344 0.370

CARW3A 0.053 0.075 0.086 0.149 0.217 0.232 0.261

ATW1A 0.189 0.215 0.229 0.296 0.374 0.389 0.423

ATW2A 0.144 0.173 0.186 0.259 0.340 0.356 0.387

AEW1A 0.253 0.279 0.294 0.373 0.456 0.474 0.508

AEW2A 0.179 0.202 0.211 0.272 0.338 0.350 0.376

AEW3A 0.110 0.125 0.131 0.170 0.215 0.224 0.243

AEW4A 0.163 0.182 0.191 0.240 0.300 0.313 0.334

TH 0.493 0.535 0.556 0.662 0.764 0.781 0.813

AE 0.163 0.188 0.201 0.270 0.353 0.370 0.404

STD Standardization

Lower .5% Lower 2.5% Lower 5% Estimate Upper 5% Upper 2.5% Upper .5%

CAR BY

CARW1A 0.327 0.341 0.348 0.388 0.426 0.433 0.449

CARW2A 0.423 0.437 0.444 0.483 0.522 0.529 0.541

CARW3A 0.432 0.448 0.455 0.495 0.538 0.545 0.558

TH BY

ATW1A 0.405 0.421 0.429 0.474 0.517 0.525 0.542

ATW2A 0.466 0.487 0.496 0.545 0.596 0.607 0.621

AE BY

AEW1A 0.432 0.455 0.466 0.517 0.569 0.579 0.596

AEW2A 0.506 0.522 0.531 0.579 0.630 0.639 0.661

AEW3A 0.457 0.470 0.476 0.510 0.550 0.556 0.572

AEW4A 0.491 0.512 0.520 0.567 0.615 0.625 0.640

AE ON

CAR 0.122 0.162 0.182 0.288 0.405 0.427 0.473

TH 0.468 0.517 0.542 0.654 0.753 0.772 0.806

TH ON

CAR 0.430 0.468 0.486 0.581 0.666 0.682 0.712

Intercepts

CARW1A 4.548 4.564 4.574 4.623 4.670 4.679 4.695

CARW2A 4.409 4.430 4.441 4.496 4.548 4.561 4.582

CARW3A 4.421 4.440 4.449 4.501 4.554 4.565 4.584

ATW1A 4.187 4.211 4.223 4.279 4.332 4.342 4.364

ATW2A 3.918 3.942 3.953 4.014 4.077 4.090 4.116

AEW1A 4.153 4.177 4.189 4.253 4.320 4.332 4.355

AEW2A 4.109 4.136 4.147 4.213 4.279 4.291 4.315

AEW3A 4.305 4.325 4.337 4.390 4.445 4.456 4.477

AEW4A 4.273 4.297 4.308 4.370 4.434 4.446 4.469

Variances

CAR 1.000 1.000 1.000 1.000 1.000 1.000 1.000

Residual Variances

CARW1A 0.047 0.055 0.059 0.089 0.144 0.153 0.175

CARW2A 0.041 0.048 0.052 0.076 0.105 0.111 0.123

CARW3A 0.015 0.021 0.025 0.043 0.065 0.070 0.079

ATW1A 0.062 0.069 0.074 0.094 0.118 0.123 0.133

ATW2A 0.061 0.072 0.077 0.104 0.134 0.140 0.150

AEW1A 0.110 0.121 0.127 0.159 0.195 0.203 0.214

AEW2A 0.079 0.088 0.093 0.125 0.168 0.176 0.193

AEW3A 0.036 0.039 0.041 0.053 0.067 0.070 0.075

AEW4A 0.064 0.072 0.077 0.102 0.134 0.141 0.152

TH 0.493 0.535 0.556 0.662 0.764 0.781 0.813

AE 0.163 0.188 0.201 0.270 0.353 0.370 0.404

CONFIDENCE INTERVALS OF TOTAL, TOTAL INDIRECT, SPECIFIC INDIRECT, AND DIRECT EFFECTS

Lower .5% Lower 2.5% Lower 5% Estimate Upper 5% Upper 2.5% Upper .5%

Effects from CAR to AE

Total 0.641 0.705 0.732 0.891 1.070 1.107 1.184

Total indirect 0.352 0.387 0.406 0.507 0.643 0.667 0.721

Specific indirect 1

AE

TH

CAR 0.352 0.387 0.406 0.507 0.643 0.667 0.721

Direct

AE

CAR 0.157 0.209 0.235 0.384 0.554 0.591 0.672

CONFIDENCE INTERVALS OF STANDARDIZED TOTAL, TOTAL INDIRECT, SPECIFIC INDIRECT, AND DIRECT EFFECTS

STDYX Standardization

Lower .5% Lower 2.5% Lower 5% Estimate Upper 5% Upper 2.5% Upper .5%

Effects from CAR to AE

Total： 0.514 0.557 0.577 0.669 0.750 0.764 0.788

Total indirect：0.281 0.305 0.317 0.380 0.466 0.486 0.518

Specific indirect 1

AE

TH

CAR 0.281 0.305 0.317 0.380 0.466 0.486 0.518

Direct

AE

CAR 0.122 0.162 0.182 0.288 0.405 0.427 0.473
