# Supplementary material for: Association between coach-athlete relationship and athlete engagement in Chinese team sports: The mediating effect of thriving
Source: PLoS One. 2023 Aug 17;18(8):e0289979. doi: 10.1371/journal.pone.0289979 (PMC10434943; doi:10.1371/journal.pone.0289979)
Supplement: S5 File — (DOCX) [file pone.0289979.s005.docx]

Mplus VERSION 8.3

MUTHEN & MUTHEN

04/11/2023 12:39 PM


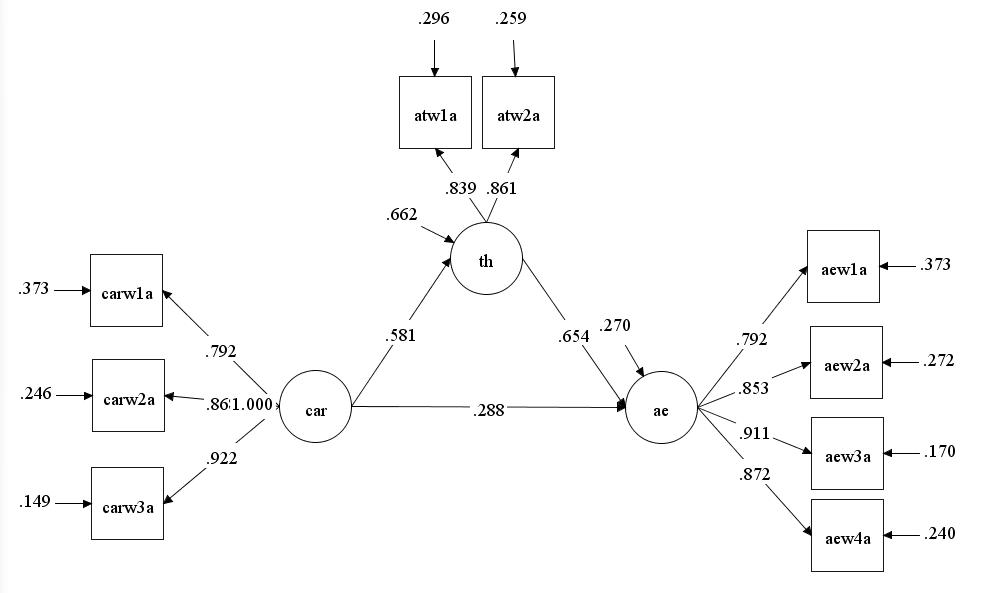


INPUT INSTRUCTIONS

DATA:

FILE IS C:\Users\DELL\Desktop\shu\+AT4+CAR3\3 CAR3-1.dat;

VARIABLE:

NAMES ARE xh gender ag syd ds grade ydxm ydnx cdnx AT1 AT2 AT3

AT4 AT5 ATw1 ATw1A AT6 AT7 AT8 AT9 AT10 ATw2 ATw2A ATsum

ATsumA CAR1 CAR5 CAR9 CAR11 CARw1 CARw1A CAR2 CAR6 CAR7

CARw2 CARw2A CAR3 CAR4 CAR8 CAR10 CARw3 CARw3A CARsum

CARsumA AE1 AE2 AE3 AE4 AEw1 AEw1A AE5 AE6 AE7 AE8 AEw2

AEw2A AE9 AE10 AE11 AE12 AEw3 AEw3A AE13 AE14 AE15 AE16

AEw4 AEw4A AEsum AEsumA;

USEVARIABLES ARE

CARw1A CARw2A CARw3A

ATw1A ATw2A

AEw1A AEw2A AEw3A AEw4A;

ANALYSIS:

TYPE=GENERAL;

ESTIMATOR=ML;

Bootstrap=5000;

MODEL:

CAR BY CARw1A CARw2A CARw3A ;

TH BY ATw1A ATw2A;

AE BY AEw1A AEw2A AEw3A AEw4A;

AE ON CAR TH;

TH ON CAR;

MODEL INDIRECT:

AE IND CAR;

OUTPUT: STANDARDIZED CINTERVAL(bcbootstrap);

INPUT READING TERMINATED NORMALLY

SUMMARY OF ANALYSIS

Number of groups 1

Number of observations 287

Number of dependent variables 9

Number of independent variables 0

Number of continuous latent variables 3

Observed dependent variables

Continuous

CARW1A CARW2A CARW3A ATW1A ATW2A AEW1A

AEW2A AEW3A AEW4A

Continuous latent variables

CAR TH AE

Estimator ML

Information matrix OBSERVED

Maximum number of iterations 1000

Convergence criterion 0.500D-04

Maximum number of steepest descent iterations 20

Number of bootstrap draws

Requested 5000

Completed 5000

Input data file(s)

C:\Users\DELL\Desktop\shu\+AT4+CAR3\3添加CAR3-1.dat

Input data format FREE

UNIVARIATE SAMPLE STATISTICS

UNIVARIATE HIGHER-ORDER MOMENT DESCRIPTIVE STATISTICS

Variable/ Mean/ Skewness/ Minimum/ % with Percentiles

Sample Size Variance Kurtosis Maximum Min/Max 20%/60% 40%/80% Median

CARW1A 4.623 -1.136 2.500 0.35% 4.000 4.750 5.000

287.000 0.240 0.657 5.000 52.26% 5.000 5.000

CARW2A 4.496 -0.820 2.667 0.70% 4.000 4.333 4.667

287.000 0.309 -0.163 5.000 43.55% 5.000 5.000

CARW3A 4.501 -0.940 2.500 0.70% 4.000 4.500 4.750

287.000 0.289 0.460 5.000 39.72% 4.750 5.000

ATW1A 4.279 -0.389 2.400 0.35% 3.800 4.000 4.200

287.000 0.318 -0.291 5.000 23.00% 4.400 5.000

ATW2A 4.014 -0.190 2.000 0.35% 3.400 3.800 4.000

287.000 0.401 -0.429 5.000 11.50% 4.200 4.600

AEW1A 4.253 -0.470 2.250 0.35% 3.750 4.000 4.000

287.000 0.426 -0.448 5.000 31.01% 4.500 5.000

AEW2A 4.213 -0.685 1.500 0.70% 3.750 4.000 4.000

287.000 0.460 0.672 5.000 28.57% 4.500 5.000

AEW3A 4.390 -0.481 2.500 0.35% 4.000 4.000 4.500

287.000 0.313 -0.435 5.000 34.84% 4.750 5.000

AEW4A 4.370 -0.890 2.250 1.05% 4.000 4.000 4.500

287.000 0.423 0.508 5.000 39.72% 4.750 5.000

THE MODEL ESTIMATION TERMINATED NORMALLY

MODEL FIT INFORMATION

Number of Free Parameters 30

Loglikelihood

H0 Value -1330.877

H1 Value -1302.792

Information Criteria

Akaike (AIC) 2721.753

Bayesian (BIC) 2831.538

Sample-Size Adjusted BIC 2736.405

(n* = (n + 2) / 24)

Chi-Square Test of Model Fit

Value 56.169

Degrees of Freedom 24

P-Value 0.0002

RMSEA (Root Mean Square Error Of Approximation)

Estimate 0.068

90 Percent C.I. 0.045 0.092

Probability RMSEA <= .05 0.092

CFI/TLI

CFI 0.983

TLI 0.975

Chi-Square Test of Model Fit for the Baseline Model

Value 1983.068

Degrees of Freedom 36

P-Value 0.0000

SRMR (Standardized Root Mean Square Residual)

Value 0.026

MODEL RESULTS

Two-Tailed

Estimate S.E. Est./S.E. P-Value

CAR BY

CARW1A 1.000 0.000 999.000 999.000

CARW2A 1.244 0.084 14.783 0.000

CARW3A 1.277 0.080 15.888 0.000

TH BY

ATW1A 1.000 0.000 999.000 999.000

ATW2A 1.152 0.073 15.850 0.000

AE BY

AEW1A 1.000 0.000 999.000 999.000

AEW2A 1.120 0.081 13.910 0.000

AEW3A 0.987 0.063 15.703 0.000

AEW4A 1.098 0.068 16.144 0.000

AE ON

CAR 0.384 0.098 3.937 0.000

TH 0.714 0.084 8.524 0.000

TH ON

CAR 0.710 0.078 9.078 0.000

Intercepts

CARW1A 4.623 0.029 158.738 0.000

CARW2A 4.496 0.033 136.208 0.000

CARW3A 4.501 0.032 141.915 0.000

ATW1A 4.279 0.033 127.999 0.000

ATW2A 4.014 0.038 105.671 0.000

AEW1A 4.253 0.039 108.755 0.000

AEW2A 4.213 0.040 105.704 0.000

AEW3A 4.390 0.033 132.472 0.000

AEW4A 4.370 0.038 114.408 0.000

Variances

CAR 0.150 0.018 8.274 0.000

Residual Variances

CARW1A 0.089 0.024 3.668 0.000

CARW2A 0.076 0.016 4.761 0.000

CARW3A 0.043 0.012 3.535 0.000

ATW1A 0.094 0.014 6.909 0.000

ATW2A 0.104 0.017 6.036 0.000

AEW1A 0.159 0.021 7.732 0.000

AEW2A 0.125 0.022 5.634 0.000

AEW3A 0.053 0.008 6.945 0.000

AEW4A 0.102 0.017 5.911 0.000

TH 0.148 0.021 6.936 0.000

AE 0.072 0.013 5.341 0.000

STANDARDIZED MODEL RESULTS

STDYX Standardization

Two-Tailed

Estimate S.E. Est./S.E. P-Value

CAR BY

CARW1A 0.792 0.050 15.807 0.000

CARW2A 0.868 0.027 32.690 0.000

CARW3A 0.922 0.022 42.584 0.000

TH BY

ATW1A 0.839 0.026 31.876 0.000

ATW2A 0.861 0.027 31.548 0.000

AE BY

AEW1A 0.792 0.031 25.161 0.000

AEW2A 0.853 0.022 38.042 0.000

AEW3A 0.911 0.014 65.305 0.000

AEW4A 0.872 0.019 45.844 0.000

AE ON

CAR 0.288 0.068 4.264 0.000

TH 0.654 0.065 10.139 0.000

TH ON

CAR 0.581 0.055 10.597 0.000

Intercepts

CARW1A 9.438 0.501 18.846 0.000

CARW2A 8.090 0.369 21.920 0.000

CARW3A 8.379 0.421 19.911 0.000

ATW1A 7.583 0.316 23.979 0.000

ATW2A 6.335 0.248 25.516 0.000

AEW1A 6.517 0.266 24.489 0.000

AEW2A 6.211 0.324 19.157 0.000

AEW3A 7.843 0.315 24.917 0.000

AEW4A 6.717 0.356 18.885 0.000

Variances

CAR 1.000 0.000 999.000 999.000

Residual Variances

CARW1A 0.373 0.078 4.766 0.000

CARW2A 0.246 0.046 5.339 0.000

CARW3A 0.149 0.040 3.752 0.000

ATW1A 0.296 0.044 6.704 0.000

ATW2A 0.259 0.047 5.544 0.000

AEW1A 0.373 0.050 7.535 0.000

AEW2A 0.272 0.038 7.110 0.000

AEW3A 0.170 0.025 6.703 0.000

AEW4A 0.240 0.033 7.259 0.000

TH 0.662 0.063 10.437 0.000

AE 0.270 0.046 5.808 0.000

STDY Standardization

Two-Tailed

Estimate S.E. Est./S.E. P-Value

CAR BY

CARW1A 0.792 0.050 15.807 0.000

CARW2A 0.868 0.027 32.690 0.000

CARW3A 0.922 0.022 42.584 0.000

TH BY

ATW1A 0.839 0.026 31.876 0.000

ATW2A 0.861 0.027 31.548 0.000

AE BY

AEW1A 0.792 0.031 25.161 0.000

AEW2A 0.853 0.022 38.042 0.000

AEW3A 0.911 0.014 65.305 0.000

AEW4A 0.872 0.019 45.844 0.000

AE ON

CAR 0.288 0.068 4.264 0.000

TH 0.654 0.065 10.139 0.000

TH ON

CAR 0.581 0.055 10.597 0.000

Intercepts

CARW1A 9.438 0.501 18.846 0.000

CARW2A 8.090 0.369 21.920 0.000

CARW3A 8.379 0.421 19.911 0.000

ATW1A 7.583 0.316 23.979 0.000

ATW2A 6.335 0.248 25.516 0.000

AEW1A 6.517 0.266 24.489 0.000

AEW2A 6.211 0.324 19.157 0.000

AEW3A 7.843 0.315 24.917 0.000

AEW4A 6.717 0.356 18.885 0.000

Variances

CAR 1.000 0.000 999.000 999.000

Residual Variances

CARW1A 0.373 0.078 4.766 0.000

CARW2A 0.246 0.046 5.339 0.000

CARW3A 0.149 0.040 3.752 0.000

ATW1A 0.296 0.044 6.704 0.000

ATW2A 0.259 0.047 5.544 0.000

AEW1A 0.373 0.050 7.535 0.000

AEW2A 0.272 0.038 7.110 0.000

AEW3A 0.170 0.025 6.703 0.000

AEW4A 0.240 0.033 7.259 0.000

TH 0.662 0.063 10.437 0.000

AE 0.270 0.046 5.808 0.000

STD Standardization

Two-Tailed

Estimate S.E. Est./S.E. P-Value

CAR BY

CARW1A 0.388 0.024 16.501 0.000

CARW2A 0.483 0.023 20.687 0.000

CARW3A 0.495 0.025 19.802 0.000

TH BY

ATW1A 0.474 0.027 17.710 0.000

ATW2A 0.545 0.031 17.678 0.000

AE BY

AEW1A 0.517 0.032 16.314 0.000

AEW2A 0.579 0.030 19.141 0.000

AEW3A 0.510 0.022 22.932 0.000

AEW4A 0.567 0.029 19.632 0.000

AE ON

CAR 0.288 0.068 4.264 0.000

TH 0.654 0.065 10.139 0.000

TH ON

CAR 0.581 0.055 10.597 0.000

Intercepts

CARW1A 4.623 0.029 158.738 0.000

CARW2A 4.496 0.033 136.208 0.000

CARW3A 4.501 0.032 141.915 0.000

ATW1A 4.279 0.033 127.999 0.000

ATW2A 4.014 0.038 105.671 0.000

AEW1A 4.253 0.039 108.755 0.000

AEW2A 4.213 0.040 105.704 0.000

AEW3A 4.390 0.033 132.472 0.000

AEW4A 4.370 0.038 114.408 0.000

Variances

CAR 1.000 0.000 999.000 999.000

Residual Variances

CARW1A 0.089 0.024 3.668 0.000

CARW2A 0.076 0.016 4.761 0.000

CARW3A 0.043 0.012 3.535 0.000

ATW1A 0.094 0.014 6.909 0.000

ATW2A 0.104 0.017 6.036 0.000

AEW1A 0.159 0.021 7.732 0.000

AEW2A 0.125 0.022 5.634 0.000

AEW3A 0.053 0.008 6.945 0.000

AEW4A 0.102 0.017 5.911 0.000

TH 0.662 0.063 10.437 0.000

AE 0.270 0.046 5.808 0.000

R-SQUARE

Observed Two-Tailed

Variable Estimate S.E. Est./S.E. P-Value

CARW1A 0.627 0.078 8.018 0.000

CARW2A 0.754 0.046 16.368 0.000

CARW3A 0.851 0.040 21.350 0.000

ATW1A 0.704 0.044 15.976 0.000

ATW2A 0.741 0.047 15.844 0.000

AEW1A 0.627 0.050 12.647 0.000

AEW2A 0.728 0.038 19.064 0.000

AEW3A 0.830 0.025 32.716 0.000

AEW4A 0.760 0.033 22.963 0.000

Latent Two-Tailed

Variable Estimate S.E. Est./S.E. P-Value

TH 0.338 0.063 5.330 0.000

AE 0.730 0.046 15.738 0.000

TOTAL, TOTAL INDIRECT, SPECIFIC INDIRECT, AND DIRECT EFFECTS

Two-Tailed

Estimate S.E. Est./S.E. P-Value

Effects from CAR to AE

Total 0.891 0.102 8.722 0.000

Total indirect 0.507 0.070 7.219 0.000

Specific indirect 1

AE

TH

CAR 0.507 0.070 7.219 0.000

Direct

AE

CAR 0.384 0.098 3.937 0.000

STANDARDIZED TOTAL, TOTAL INDIRECT, SPECIFIC INDIRECT, AND DIRECT EFFECTS

STDYX Standardization

Two-Tailed

Estimate S.E. Est./S.E. P-Value

Effects from CAR to AE

Total 0.669 0.053 12.596 0.000

Total indirect 0.380 0.045 8.474 0.000

Specific indirect 1

AE

TH

CAR 0.380 0.045 8.474 0.000

Direct

AE

CAR 0.288 0.068 4.264 0.000

STDY Standardization

Two-Tailed

Estimate S.E. Est./S.E. P-Value

Effects from CAR to AE

Total 0.669 0.053 12.596 0.000

Total indirect 0.380 0.045 8.474 0.000

Specific indirect 1

AE

TH

CAR 0.380 0.045 8.474 0.000

Direct

AE

CAR 0.288 0.068 4.264 0.000

STD Standardization

Two-Tailed

Estimate S.E. Est./S.E. P-Value

Effects from CAR to AE

Total 0.669 0.053 12.596 0.000

Total indirect 0.380 0.045 8.474 0.000

Specific indirect 1

AE

TH

CAR 0.380 0.045 8.474 0.000

Direct

AE

CAR 0.288 0.068 4.264 0.000

CONFIDENCE INTERVALS OF MODEL RESULTS

Lower .5% Lower 2.5% Lower 5% Estimate Upper 5% Upper 2.5% Upper .5%

CAR BY

CARW1A 1.000 1.000 1.000 1.000 1.000 1.000 1.000

CARW2A 1.055 1.095 1.120 1.244 1.397 1.427 1.500

CARW3A 1.101 1.141 1.160 1.277 1.423 1.457 1.520

TH BY

ATW1A 1.000 1.000 1.000 1.000 1.000 1.000 1.000

ATW2A 0.985 1.021 1.041 1.152 1.282 1.307 1.368

AE BY

AEW1A 1.000 1.000 1.000 1.000 1.000 1.000 1.000

AEW2A 0.940 0.980 1.000 1.120 1.264 1.297 1.365

AEW3A 0.845 0.876 0.893 0.987 1.099 1.122 1.178

AEW4A 0.931 0.974 0.991 1.098 1.211 1.235 1.295

AE ON

CAR 0.157 0.209 0.235 0.384 0.554 0.591 0.672

TH 0.493 0.551 0.579 0.714 0.852 0.880 0.937

TH ON

CAR 0.517 0.563 0.587 0.710 0.842 0.871 0.932

Intercepts

CARW1A 4.548 4.564 4.574 4.623 4.670 4.679 4.695

CARW2A 4.409 4.430 4.441 4.496 4.548 4.561 4.582

CARW3A 4.421 4.440 4.449 4.501 4.554 4.565 4.584

ATW1A 4.187 4.211 4.223 4.279 4.332 4.342 4.364

ATW2A 3.918 3.942 3.953 4.014 4.077 4.090 4.116

AEW1A 4.153 4.177 4.189 4.253 4.320 4.332 4.355

AEW2A 4.109 4.136 4.147 4.213 4.279 4.291 4.315

AEW3A 4.305 4.325 4.337 4.390 4.445 4.456 4.477

AEW4A 4.273 4.297 4.308 4.370 4.434 4.446 4.469

Variances

CAR 0.107 0.116 0.121 0.150 0.181 0.188 0.202

Residual Variances

CARW1A 0.047 0.055 0.059 0.089 0.144 0.153 0.175

CARW2A 0.041 0.048 0.052 0.076 0.105 0.111 0.123

CARW3A 0.015 0.021 0.025 0.043 0.065 0.070 0.079

ATW1A 0.062 0.069 0.074 0.094 0.118 0.123 0.133

ATW2A 0.061 0.072 0.077 0.104 0.134 0.140 0.150

AEW1A 0.110 0.121 0.127 0.159 0.195 0.203 0.214

AEW2A 0.079 0.088 0.093 0.125 0.168 0.176 0.193

AEW3A 0.036 0.039 0.041 0.053 0.067 0.070 0.075

AEW4A 0.064 0.072 0.077 0.102 0.134 0.141 0.152

TH 0.100 0.111 0.116 0.148 0.187 0.196 0.210

AE 0.043 0.049 0.053 0.072 0.098 0.102 0.113

CONFIDENCE INTERVALS OF STANDARDIZED MODEL RESULTS

STDYX Standardization

Lower .5% Lower 2.5% Lower 5% Estimate Upper 5% Upper 2.5% Upper .5%

CAR BY

CARW1A 0.632 0.668 0.689 0.792 0.859 0.868 0.886

CARW2A 0.793 0.810 0.822 0.868 0.908 0.915 0.928

CARW3A 0.860 0.876 0.885 0.922 0.956 0.962 0.973

TH BY

ATW1A 0.760 0.782 0.791 0.839 0.878 0.886 0.901

ATW2A 0.782 0.803 0.813 0.861 0.902 0.909 0.925

AE BY

AEW1A 0.699 0.725 0.737 0.792 0.840 0.849 0.864

AEW2A 0.789 0.806 0.814 0.853 0.888 0.893 0.906

AEW3A 0.869 0.881 0.886 0.911 0.932 0.936 0.943

AEW4A 0.816 0.829 0.836 0.872 0.899 0.904 0.915

AE ON

CAR 0.122 0.162 0.182 0.288 0.405 0.427 0.473

TH 0.468 0.517 0.542 0.654 0.753 0.772 0.806

TH ON

CAR 0.430 0.468 0.486 0.581 0.666 0.682 0.712

Intercepts

CARW1A 8.210 8.492 8.635 9.438 10.282 10.461 10.774

CARW2A 7.237 7.412 7.513 8.090 8.726 8.872 9.130

CARW3A 7.397 7.621 7.727 8.379 9.088 9.246 9.553

ATW1A 6.815 6.977 7.074 7.583 8.115 8.224 8.409

ATW2A 5.729 5.869 5.938 6.335 6.750 6.843 6.997

AEW1A 5.898 6.024 6.091 6.517 6.976 7.063 7.236

AEW2A 5.421 5.602 5.681 6.211 6.740 6.844 7.037

AEW3A 7.054 7.203 7.291 7.843 8.327 8.441 8.611

AEW4A 5.901 6.074 6.163 6.717 7.330 7.459 7.739

Variances

CAR 1.000 1.000 1.000 1.000 1.000 1.000 1.000

Residual Variances

CARW1A 0.215 0.247 0.263 0.373 0.526 0.553 0.600

CARW2A 0.139 0.162 0.176 0.246 0.325 0.344 0.370

CARW3A 0.053 0.075 0.086 0.149 0.217 0.232 0.261

ATW1A 0.189 0.215 0.229 0.296 0.374 0.389 0.423

ATW2A 0.144 0.173 0.186 0.259 0.340 0.356 0.387

AEW1A 0.253 0.279 0.294 0.373 0.456 0.474 0.508

AEW2A 0.179 0.202 0.211 0.272 0.338 0.350 0.376

AEW3A 0.110 0.125 0.131 0.170 0.215 0.224 0.243

AEW4A 0.163 0.182 0.191 0.240 0.300 0.313 0.334

TH 0.493 0.535 0.556 0.662 0.764 0.781 0.813

AE 0.163 0.188 0.201 0.270 0.353 0.370 0.404

STDY Standardization

Lower .5% Lower 2.5% Lower 5% Estimate Upper 5% Upper 2.5% Upper .5%

CAR BY

CARW1A 0.632 0.668 0.689 0.792 0.859 0.868 0.886

CARW2A 0.793 0.810 0.822 0.868 0.908 0.915 0.928

CARW3A 0.860 0.876 0.885 0.922 0.956 0.962 0.973

TH BY

ATW1A 0.760 0.782 0.791 0.839 0.878 0.886 0.901

ATW2A 0.782 0.803 0.813 0.861 0.902 0.909 0.925

AE BY

AEW1A 0.699 0.725 0.737 0.792 0.840 0.849 0.864

AEW2A 0.789 0.806 0.814 0.853 0.888 0.893 0.906

AEW3A 0.869 0.881 0.886 0.911 0.932 0.936 0.943

AEW4A 0.816 0.829 0.836 0.872 0.899 0.904 0.915

AE ON

CAR 0.122 0.162 0.182 0.288 0.405 0.427 0.473

TH 0.468 0.517 0.542 0.654 0.753 0.772 0.806

TH ON

CAR 0.430 0.468 0.486 0.581 0.666 0.682 0.712

Intercepts

CARW1A 8.210 8.492 8.635 9.438 10.282 10.461 10.774

CARW2A 7.237 7.412 7.513 8.090 8.726 8.872 9.130

CARW3A 7.397 7.621 7.727 8.379 9.088 9.246 9.553

ATW1A 6.815 6.977 7.074 7.583 8.115 8.224 8.409

ATW2A 5.729 5.869 5.938 6.335 6.750 6.843 6.997

AEW1A 5.898 6.024 6.091 6.517 6.976 7.063 7.236

AEW2A 5.421 5.602 5.681 6.211 6.740 6.844 7.037

AEW3A 7.054 7.203 7.291 7.843 8.327 8.441 8.611

AEW4A 5.901 6.074 6.163 6.717 7.330 7.459 7.739

Variances

CAR 1.000 1.000 1.000 1.000 1.000 1.000 1.000

Residual Variances

CARW1A 0.215 0.247 0.263 0.373 0.526 0.553 0.600

CARW2A 0.139 0.162 0.176 0.246 0.325 0.344 0.370

CARW3A 0.053 0.075 0.086 0.149 0.217 0.232 0.261

ATW1A 0.189 0.215 0.229 0.296 0.374 0.389 0.423

ATW2A 0.144 0.173 0.186 0.259 0.340 0.356 0.387

AEW1A 0.253 0.279 0.294 0.373 0.456 0.474 0.508

AEW2A 0.179 0.202 0.211 0.272 0.338 0.350 0.376

AEW3A 0.110 0.125 0.131 0.170 0.215 0.224 0.243

AEW4A 0.163 0.182 0.191 0.240 0.300 0.313 0.334

TH 0.493 0.535 0.556 0.662 0.764 0.781 0.813

AE 0.163 0.188 0.201 0.270 0.353 0.370 0.404

STD Standardization

Lower .5% Lower 2.5% Lower 5% Estimate Upper 5% Upper 2.5% Upper .5%

CAR BY

CARW1A 0.327 0.341 0.348 0.388 0.426 0.433 0.449

CARW2A 0.423 0.437 0.444 0.483 0.522 0.529 0.541

CARW3A 0.432 0.448 0.455 0.495 0.538 0.545 0.558

TH BY

ATW1A 0.405 0.421 0.429 0.474 0.517 0.525 0.542

ATW2A 0.466 0.487 0.496 0.545 0.596 0.607 0.621

AE BY

AEW1A 0.432 0.455 0.466 0.517 0.569 0.579 0.596

AEW2A 0.506 0.522 0.531 0.579 0.630 0.639 0.661

AEW3A 0.457 0.470 0.476 0.510 0.550 0.556 0.572

AEW4A 0.491 0.512 0.520 0.567 0.615 0.625 0.640

AE ON

CAR 0.122 0.162 0.182 0.288 0.405 0.427 0.473

TH 0.468 0.517 0.542 0.654 0.753 0.772 0.806

TH ON

CAR 0.430 0.468 0.486 0.581 0.666 0.682 0.712

Intercepts

CARW1A 4.548 4.564 4.574 4.623 4.670 4.679 4.695

CARW2A 4.409 4.430 4.441 4.496 4.548 4.561 4.582

CARW3A 4.421 4.440 4.449 4.501 4.554 4.565 4.584

ATW1A 4.187 4.211 4.223 4.279 4.332 4.342 4.364

ATW2A 3.918 3.942 3.953 4.014 4.077 4.090 4.116

AEW1A 4.153 4.177 4.189 4.253 4.320 4.332 4.355

AEW2A 4.109 4.136 4.147 4.213 4.279 4.291 4.315

AEW3A 4.305 4.325 4.337 4.390 4.445 4.456 4.477

AEW4A 4.273 4.297 4.308 4.370 4.434 4.446 4.469

Variances

CAR 1.000 1.000 1.000 1.000 1.000 1.000 1.000

Residual Variances

CARW1A 0.047 0.055 0.059 0.089 0.144 0.153 0.175

CARW2A 0.041 0.048 0.052 0.076 0.105 0.111 0.123

CARW3A 0.015 0.021 0.025 0.043 0.065 0.070 0.079

ATW1A 0.062 0.069 0.074 0.094 0.118 0.123 0.133

ATW2A 0.061 0.072 0.077 0.104 0.134 0.140 0.150

AEW1A 0.110 0.121 0.127 0.159 0.195 0.203 0.214

AEW2A 0.079 0.088 0.093 0.125 0.168 0.176 0.193

AEW3A 0.036 0.039 0.041 0.053 0.067 0.070 0.075

AEW4A 0.064 0.072 0.077 0.102 0.134 0.141 0.152

TH 0.493 0.535 0.556 0.662 0.764 0.781 0.813

AE 0.163 0.188 0.201 0.270 0.353 0.370 0.404

CONFIDENCE INTERVALS OF TOTAL, TOTAL INDIRECT, SPECIFIC INDIRECT, AND DIRECT EFFECTS

Lower .5% Lower 2.5% Lower 5% Estimate Upper 5% Upper 2.5% Upper .5%

Effects from CAR to AE

Total 0.641 0.705 0.732 0.891 1.070 1.107 1.184

Total indirect 0.352 0.387 0.406 0.507 0.643 0.667 0.721

Specific indirect 1

AE

TH

CAR 0.352 0.387 0.406 0.507 0.643 0.667 0.721

Direct

AE

CAR 0.157 0.209 0.235 0.384 0.554 0.591 0.672

CONFIDENCE INTERVALS OF STANDARDIZED TOTAL, TOTAL INDIRECT, SPECIFIC INDIRECT, AND DIRECT EFFECTS

STDYX Standardization

Lower .5% Lower 2.5% Lower 5% Estimate Upper 5% Upper 2.5% Upper .5%

Effects from CAR to AE

Total： 0.514 0.557 0.577 0.669 0.750 0.764 0.788

Total indirect：0.281 0.305 0.317 0.380 0.466 0.486 0.518

Specific indirect 1

AE

TH

CAR 0.281 0.305 0.317 0.380 0.466 0.486 0.518

Direct

AE

CAR 0.122 0.162 0.182 0.288 0.405 0.427 0.473

STDY Standardization

Lower .5% Lower 2.5% Lower 5% Estimate Upper 5% Upper 2.5% Upper .5%

Effects from CAR to AE

Total 0.514 0.557 0.577 0.669 0.750 0.764 0.788

Total indirect 0.281 0.305 0.317 0.380 0.466 0.486 0.518

Specific indirect 1

AE

TH

CAR 0.281 0.305 0.317 0.380 0.466 0.486 0.518

Direct

AE

CAR 0.122 0.162 0.182 0.288 0.405 0.427 0.473

STD Standardization

Lower .5% Lower 2.5% Lower 5% Estimate Upper 5% Upper 2.5% Upper .5%

Effects from CAR to AE

Total 0.514 0.557 0.577 0.669 0.750 0.764 0.788

Total indirect 0.281 0.305 0.317 0.380 0.466 0.486 0.518

Specific indirect 1

AE

TH

CAR 0.281 0.305 0.317 0.380 0.466 0.486 0.518

Direct

AE

CAR 0.122 0.162 0.182 0.288 0.405 0.427 0.473

DIAGRAM INFORMATION

Use View Diagram under the Diagram menu in the Mplus Editor to view the diagram.

If running Mplus from the Mplus Diagrammer, the diagram opens automatically.

Diagram output

c:\users\dell\desktop\数据\原始数据\mptext3.dgm

Beginning Time: 12:39:40

Ending Time: 12:39:50

Elapsed Time: 00:00:10

MUTHEN & MUTHEN

3463 Stoner Ave.

Los Angeles, CA 90066

Tel: (310) 391-9971

Fax: (310) 391-8971

Web: www.StatModel.com

Support: Support@StatModel.com

Copyright (c) 1998-2019 Muthen & Muthen
