# Supplementary material for: Association between coach-athlete relationship and athlete engagement in Chinese team sports: The mediating effect of thriving
Source: PLoS One. 2023 Aug 17;18(8):e0289979. doi: 10.1371/journal.pone.0289979 (PMC10434943; doi:10.1371/journal.pone.0289979)
Supplement: S7 File — (DOCX) [file pone.0289979.s007.docx]

**Appendix A. Original Questionnaire**

| Coach-Athlete Relationship dimension and items | | | | | |
| --- | --- | --- | --- | --- | --- |
| **Items** | **strongly disagree** | **disagree** | **not sure** | **agree** | **strongly agree** |
| 1. I feel close to my coach. | 1 | 2 | 3 | 4 | 5 |
| 2. I feel committed to my coach. | 1 | 2 | 3 | 4 | 5 |
| 3. I feel that my sport career is promising with my coach. | 1 | 2 | 3 | 4 | 5 |
| 4. I like my coach. | 1 | 2 | 3 | 4 | 5 |
| 5. I trust my coach. | 1 | 2 | 3 | 4 | 5 |
| 6. I respect my coach. | 1 | 2 | 3 | 4 | 5 |
| 7. I feel appreciation for the sacriﬁces my coach has experienced in order to improve his performance. | 1 | 2 | 3 | 4 | 5 |
| 8. When I am coached by my coach, I feel at ease. | 1 | 2 | 3 | 4 | 5 |
| 9. When I am coached by my coach, I feel responsive to his efforts. | 1 | 2 | 3 | 4 | 5 |
| 10. When I am coached by my coach, I am ready to do my best. | 1 | 2 | 3 | 4 | 5 |
| 11. When I am coached by my coach, I adopt a friendly stance. | 1 | 2 | 3 | 4 | 5 |

| Thriving dimension and items | | | | | |
| --- | --- | --- | --- | --- | --- |
| **Items** | **strongly disagree** | **disagree** | **not sure** | **agree** | **strongly agree** |
| 1. In sports training and competition, I find myself learning often. | 1 | 2 | 3 | 4 | 5 |
| 1. In sports training and competition, I continue to learn more and more as time goes by. | 1 | 2 | 3 | 4 | 5 |
| 1. In sports training and competition, I see myself continually improving. | 1 | 2 | 3 | 4 | 5 |
| 1. In sports training and competition, I am not learning (R). | 1 | 2 | 3 | 4 | 5 |
| 1. In sports training and competition, I have developed a lot as a person. | 1 | 2 | 3 | 4 | 5 |
| 1. In sports training and competition, I feel alive and vital. | 1 | 2 | 3 | 4 | 5 |
| 1. In sports training and competition, I have energy and spirit. | 1 | 2 | 3 | 4 | 5 |
| 1. In sports training and competition, I do not feel very energetic (R). | 1 | 2 | 3 | 4 | 5 |
| 1. In sports training and competition, I feel alert and awake. | 1 | 2 | 3 | 4 | 5 |
| 1. In sports training and competition, I am looking forward to each new day. | 1 | 3 | 4 | 4 | 5 |

| Athlete Engagement dimension and items | | | | | |
| --- | --- | --- | --- | --- | --- |
| **Items** | **strongly disagree** | **disagree** | **not sure** | **agree** | **strongly agree** |
| 1. I believe I am capable of accomplishing my goals in sport. | 1 | 2 | 3 | 4 | 5 |
| 2. I am dedicated to achieving my goals in sport. | 1 | 2 | 3 | 4 | 5 |
| 3. I feel excited about my sport. | 1 | 2 | 3 | 4 | 5 |
| 4. I feel energized when I participate in my sport. | 1 | 2 | 3 | 4 | 5 |
| 5. I feel capable of success in my sport. | 1 | 2 | 3 | 4 | 5 |
| 6. I am determined to achieve my goals in sport. | 1 | 2 | 3 | 4 | 5 |
| 7. I am enthusiastic about my sport. | 1 | 2 | 3 | 4 | 5 |
| 8. I feel energetic when I participate in my sport. | 1 | 2 | 3 | 4 | 5 |
| 9. I believe I have the skills/technique to be successful in my sport. | 1 | 2 | 3 | 4 | 5 |
| 10. I am devoted to my sport. | 1 | 2 | 3 | 4 | 5 |
| 11. I enjoy my sport. | 1 | 2 | 3 | 4 | 5 |
| 12. I feel really alive when I participate in my sport. | 1 | 2 | 3 | 4 | 5 |
| 13. I am confident in my abilities. | 1 | 2 | 3 | 4 | 5 |
| 14. I want to work hard to achieve my goals in sport. | 1 | 2 | 3 | 4 | 5 |
| 15. I have fun in my sport. | 1 | 2 | 3 | 4 | 5 |
| 16. I feel mentally alert when I participate in my sport. | 1 | 2 | 3 | 4 | 5 |

**Appendix B. The actual Chinese questionnaire used in this study**

**运动员调查问卷**

| 尊敬的运动员，感谢参与本次调研。这份问卷是由浙江师范大学科研小组设计。本次调査是匿名调查，所有资料仅作科学研究之用，无需担心信息泄露。请仔细阅读指导语，认真、准确地回答每个问题。 | | |
| --- | --- | --- |
| 性别： 男🞎 女🞎 出生日期： 年 月 |  |  |
| 生源地： 城市🞎 农村🞎 是否为独生子女： 是🞎 否🞎 |  |  |
| 年级： 大一🞎 大二🞎 大三🞎 大四🞎 研究生🞎 其他🞎 |  |  |
| 运动项目： 运动训练年限： 年 |  |  |
| 参加大学运动队年限： 1年以内🞎 1年🞎 2年🞎 3年🞎 4年及以上🞎 |  |  |
| 运动等级： 三级🞎 二级🞎 一级🞎 健将🞎 国际健将🞎 不确定🞎  比赛级别： 国家级比赛🞎 省级比赛🞎 地区或市县级比赛🞎 不确定🞎  最好成绩： 名次 |  |  |

| 题  号 | 指导语：以下内容是为了解您**在运动情境中**的反应情况。根据句子的内容与自己的实际情况相符合的程度，在相应的数字上打√。 | **完全不同意** | **不**  **同意** | **不确定** | **比较同意** | **完全同意** |
| --- | --- | --- | --- | --- | --- | --- |
| 1 | 我相信我能实现自己的运动目标。 | 1 | 2 | 3 | 4 | 5 |
| 2 | 我认为自己有能力在运动中获得成功。 | 1 | 2 | 3 | 4 | 5 |
| 3 | 我相信自己拥有获得运动成功所需的技能和技巧。 | 1 | 2 | 3 | 4 | 5 |
| 4 | 我对自己的运动能力很有信心。 | 1 | 2 | 3 | 4 | 5 |
| 5 | 我能精力充沛地参加我所从事的运动项目。 | 1 | 2 | 3 | 4 | 5 |
| 6 | 只要是从事我所参加的运动项目，我就感到自己充满活力。 | 1 | 2 | 3 | 4 | 5 |
| 7 | 只要是从事我所参加的运动项目，我就能感受到自己活跃的生命力。 | 1 | 2 | 3 | 4 | 5 |
| 8 | 当我参加的是我所从事的运动项目时，我的思维会非常敏捷。 | 1 | 2 | 3 | 4 | 5 |
| 9 | 我将会致力于实现我的运动目标。 | 1 | 2 | 3 | 4 | 5 |
| 10 | 我下定决心要实现我的运动目标。 | 1 | 2 | 3 | 4 | 5 |
| 11 | 我钟爱我自己所从事的体育运动项目。 | 1 | 2 | 3 | 4 | 5 |
| 12 | 我会通过努力奋斗来实现自己的运动目标。 | 1 | 2 | 3 | 4 | 5 |
| 13 | 我对我所从事的运动项目感到兴奋。 | 1 | 2 | 3 | 4 | 5 |
| 14 | 我对我所从事的运动项目充满热情。 | 1 | 2 | 3 | 4 | 5 |
| 15 | 当从事我所参加的运动项目时，我很享受。 | 1 | 2 | 3 | 4 | 5 |
| 16 | 我认为我所参加的运动项目很有乐趣。 | 1 | 2 | 3 | 4 | 5 |
| ——**感谢您的配合！祝君生活愉快！**—— | | | | | | |

| 题  号 | 指导语：根据个人的**训练状态及运动经历**，选择一个最符合自己实际情况的选项，请在相应的数字上打√。 | **完全不同意** | **不同意** | **不确定** | **比较同意** | **完全同意** |
| --- | --- | --- | --- | --- | --- | --- |
| 18 | 在运动训练中，我时常可以学到新的知识或技能。 | 1 | 2 | 3 | 4 | 5 |
| 19 | 尽管我已有些参训年限，但我还是能从训练中，持续学习到或感悟到新的知识或技巧。 | 1 | 2 | 3 | 4 | 5 |
| 20 | 我认为自己的运动水平还在进一步提高。 | 1 | 2 | 3 | 4 | 5 |
| 21 | 在训练中，我感到自己已经学不到什么新东西了。 | 1 | 2 | 3 | 4 | 5 |
| 22 | 在运动训练中，我还收获了很多运动能力以外的提升，如为人、为学等。 | 1 | 2 | 3 | 4 | 5 |
| 23 | 训练时，我感到生机勃勃。 | 1 | 2 | 3 | 4 | 5 |
| 24 | 训练时，我感到充满能量和精力感。 | 1 | 2 | 3 | 4 | 5 |
| 25 | 训练时，我感到自己提不起劲来。 | 1 | 2 | 3 | 4 | 5 |
| 26 | 训练时，我感到自己专注且投入。 | 1 | 2 | 3 | 4 | 5 |
| 27 | 我期待着每一天运动训练的到来。 | 1 | 2 | 3 | 4 | 5 |
|  | | | | | | |
|  | 指导语：以下语句描述了个体的一些**情况与看法**。请仔细阅读后，根据句子的内容与自己的实际情况相符合的程度，在相应的数字上打√。 | **完全不同意** | **不同意** | **不确定** | **比较同意** | **完全同意** |
| 29 | 我喜欢我的教练。 | 1 | 2 | 3 | 4 | 5 |
| 30 | 我与教练的关系比较密切。 | 1 | 2 | 3 | 4 | 5 |
| 31 | 当教练指导我时，我会作出积极冋应。 | 1 | 2 | 3 | 4 | 5 |
| 32 | 当教练指导我时，我表现得比较好。 | 1 | 2 | 3 | 4 | 5 |
| 33 | 我信任我的教练。 | 1 | 2 | 3 | 4 | 5 |
| 34 | 我忠于我的教练，我愿意和他保持长期的合作关系。 | 1 | 2 | 3 | 4 | 5 |
| 35 | 跟我的教练在一起，我感到我的运动生涯充满了希望。 | 1 | 2 | 3 | 4 | 5 |
| 36 | 当教练指导我时，我觉得轻松。 | 1 | 2 | 3 | 4 | 5 |
| 37 | 我尊重我的教练。 | 1 | 2 | 3 | 4 | 5 |
| 38 | 当教练指导我时，我会全力以赴，力争做到最好。 | 1 | 2 | 3 | 4 | 5 |
| 39 | 我很感激教练为了提高我的运动成绩所作出的牺牲。 | 1 | 2 | 3 | 4 | 5 |

——**感谢您的配合！祝君生活愉快！**——
